# A SEPT1-based scaffold is required for Golgi integrity and function

**DOI:** 10.1242/jcs.225557

**Published:** 2019-02-01

**Authors:** Kyungyeun Song, Claudia Gras, Gabrielle Capin, Niclas Gimber, Martin Lehmann, Saif Mohd, Dmytro Puchkov, Maria Rödiger, Ilka Wilhelmi, Oliver Daumke, Jan Schmoranzer, Annette Schürmann, Michael Krauss

**Affiliations:** 1 Leibniz-Forschungsinstitut für Molekulare Pharmakologie (FMP) Berlin, Molecular Pharmacology and Cell Biology, 13125 Berlin, Germany; 2 Charité Universitätsmedizin Berlin, Advanced Medical Bioimaging Core Facility - AMBIO, 10117 Berlin, Germany; 3 Leibniz-Forschungsinstitut für Molekulare Pharmakologie (FMP) Berlin, Cellular Imaging Facility, 13125 Berlin, Germany; 4 Max-Delmbrück-Centrum für Molekulare Medizin, 13125 Berlin, Germany; 5 Deutsches Institut für Ernährungsforschung, Potsdam Rehbrücke, and German Center for Diabetes Research (DZD), München-Neuherberg, 14558 Potsdam-Rehbrücke, Germany

**Keywords:** Septin, SEPT1, GM130, CEP170, Microtubule nucleation, Golgi

## Abstract

Compartmentalization of membrane transport and signaling processes is of pivotal importance to eukaryotic cell function. While plasma membrane compartmentalization and dynamics are well known to depend on the scaffolding function of septin GTPases, the roles of septins at intracellular membranes have remained largely elusive. Here, we show that the structural and functional integrity of the Golgi depends on its association with a septin 1 (SEPT1)-based scaffold, which promotes local microtubule nucleation and positioning of the Golgi. SEPT1 function depends on the Golgi matrix protein GM130 (also known as GOLGA2) and on centrosomal proteins, including CEP170 and components of γ-tubulin ring complex (γ-Turc), to facilitate the perinuclear concentration of Golgi membranes. Accordingly, SEPT1 depletion triggers a massive fragmentation of the Golgi ribbon, thereby compromising anterograde membrane traffic at the level of the Golgi.

## Abstract (precis)

**Highlighted Article:** SEPT1 associates with the Golgi complex through GM130, and is required for maintaining Golgi architecture and function by connecting *cis*-Golgi membranes to the microtubule-nucleating machinery.

## INTRODUCTION

In mammalian interphase cells the Golgi is localized in the perinuclear area, in close proximity to the centrosome, where it forms a ribbon-like system of closely apposed cisternae, which are polarized in a *cis*-to-*trans* fashion (Nakamura et al., 2012). The Golgi serves as a major membrane trafficking hub, where anterograde and retrograde transport routes meet (Brandizzi and Barlowe, 2013; Guo et al., 2014; Progida and Bakke, 2016). *De novo*-synthesized polypeptides are delivered from the endoplasmic reticulum (ER) to early Golgi compartments, progress through the Golgi stacks and are eventually packed into post-Golgi carriers at late cisternae. Conversely, the *trans*-Golgi receives material from endosomes for delivery to the *cis*-Golgi and the ER. This mechanism allows for the retrieval of mis-localized proteins that have escaped the intracellular sorting machinery, but also enables bacterial toxins, like cholera toxin, to reach their destination in the cytoplasm. The longitudinal and lateral association of individual cisternae within the Golgi ribbon, and the tethering of transport vesicles to the Golgi are mediated by Golgi matrix proteins, including peripherally associated Golgi reassembly stacking proteins (GRASPs) and members of the golgin family (Ramirez and Lowe, 2009; Wong and Munro, 2014).

The structural organization, positioning and function of the Golgi depend on the integrity of cytoskeletal elements (Gurel et al., 2014), but to date the functional interplay between different components is poorly understood. Actin microfilaments have been shown to stabilize the Golgi ribbon (Egea et al., 2013). Accordingly, actin regulatory and actin-associated proteins such as Rho GTPases, formins or β3-spectrin are required to maintain cisternal integrity, and several of them are essential for transport to, through or out of the Golgi (Egea et al., 2013; Kage et al., 2017; Long and Simpson, 2017; Salcedo-Sicilia et al., 2013).

The Golgi also serves as a microtubule nucleation center, and this local microtubule nucleation is required to support Golgi integrity and directionality of post-Golgi trafficking in many cell types (Sanders and Kaverina, 2015). Several Golgi-associated factors have been implicated in this process. A-kinase anchoring protein 9 (AKAP450, also known as AKAP9), for instance, associates with the *cis*-Golgi through the golgin GM130 (also known as GOLGA2), where it acts in concert with myomegalin to recruit the γ-tubulin ring complex (γ-Turc) (Rivero et al., 2009; Wang et al., 2014). By recruiting microtubule end-binding proteins, myomegalin also regulates microtubule minus-end organization (Yang et al., 2017). At the *trans*-side of the Golgi, on the other hand, cytoplasmic linker-associated proteins (CLASPs) are recruited by the golgin GCC185 (also known as GCC2) to promote the formation of asymmetric microtubules and to control polarized trafficking (Efimov et al., 2007; Miller et al., 2009). CLASPs thereby mediate the association of α-tubulin–β-tubulin dimers to support microtubule rescue and regulate the localization of microtubule plus-end-binding proteins (Al-Bassam et al., 2010; Grimaldi et al., 2014; Yang et al., 2017).

Septins constitute a family of GTP-binding proteins that assemble into filaments via nucleotide-dependent interactions between the GTPase domains (Sirajuddin et al., 2007) and are, therefore, considered as a fourth component of the cytoskeleton (Mostowy and Cossart, 2012). Septin filaments associate with bent and negatively charged membrane surfaces (Bridges et al., 2016; Tanaka-Takiguchi et al., 2009), where they act as scaffolds to stabilize membrane curvature and to recruit septin-specific effector proteins (Kinoshita, 2006). Based on these properties, septins have been implicated in several membrane-dependent events, and are believed to predominantly act at the plasma membrane, for instance during cell division, membrane traffic or signaling (Fung et al., 2014; Mostowy and Cossart, 2012). However, it is largely unclear how septins might contribute to the organization of intracellular organelles, such as the Golgi. Also, little is known about the molecular machineries that support septin functions, as only few non-septin binding partners have been identified to date.

Here, we elucidate a novel function of septins in maintaining Golgi positioning, architecture and function. We show that SEPT1 is organized in filament-like structures aligning predominantly with *cis*-Golgi cisternae in a GM130-dependent manner. Its depletion triggers massive scattering of the Golgi, a dilation of Golgi cisternae, and impairs anterograde and retrograde membrane traffic from and to the Golgi. We further demonstrate that SEPT1 forms an endogenous complex with components of γ-Turc and centrosomal protein 170 (CEP170). The latter is recruited to the Golgi in a SEPT1-dependent manner. Thereby, SEPT1 supports the nucleation of Golgi-derived microtubules, and allows for efficient secretory membrane traffic.

## RESULTS

### SEPT1 localizes to the Golgi

**Fig. 1.**
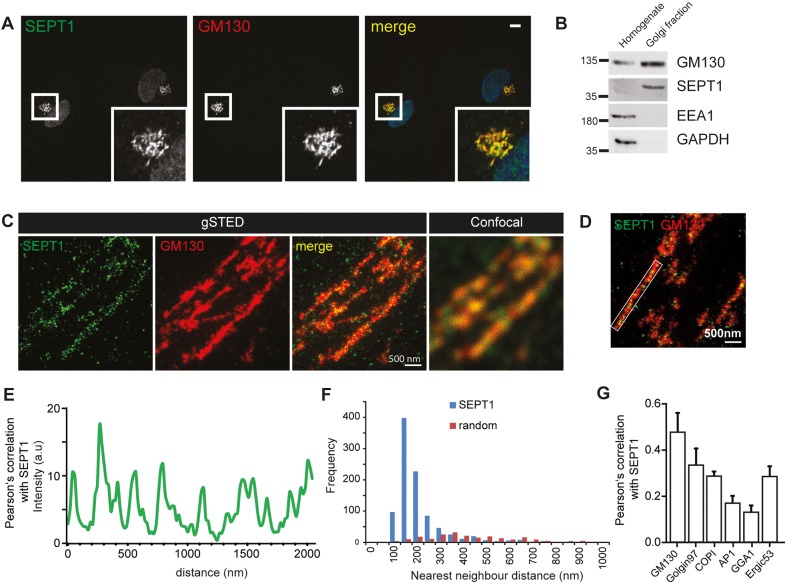
**SEPT1 localizes predominantly to *cis*-Golgi membranes.** (A) HeLa cells were fixed with 2% PFA and immunolabeled for SEPT1 and GM130. Scale bar: 10 µm. See also Fig. S1. (B) SEPT1 is enriched in the Golgi-fraction of HeLa cells (GM130, Golgi; EEA1, endosomes; GAPDH, cytosol). (C) gSTED microscopy analysis of HeLa cells co-immunolabeled with antibodies recognizing SEPT1 and GM130 (*cis*-Golgi). Scale bar: 500 nm. See also Fig. S2. (D,E) Line scan of SEPT1 localizations along Golgi membranes (D), and fluorescence intensity profile (E). (F) Nearest neighbor analysis of SEPT1 localizations. Histogram depicting the frequencies of distances observed along Golgi membranes (blue bars) or within the nucleus (unspecific staining, red bars). (G) Pearson's correlation coefficients determined from STED microscopy images upon staining for SEPT1 and various Golgi marker proteins (*n*=10–11). GM130, 0.47; Golgin 97, 0.33; COPI, 0.29; AP-1, 0.17; GGA1, 0.13; ERGIC53, 0.28.

Septins comprise a family of filament-forming GTPases with well-characterized roles in many pivotal processes, including cytokinesis, membrane trafficking and signaling. Based on their well-characterized association with phosphatidylinositol 4,5-bisphosphate (Song et al., 2016), septins are believed to exert these functions, in most cases, at the level of the plasma membrane. However, little is known about the association of septins with the endomembrane system. To unravel such putative functions, we screened a collection of tagged septin isoforms for their localization in HeLa cells. To our surprise Myc-tagged SEPT1 displayed a prominent perinuclear localization (Fig. S1A), a finding that could be confirmed with an antibody detecting endogenous SEPT1 in a variety of cell types, including HeLa, RPE1 and Jurkat cells (Fig. 1A; Fig. S1B,C). The majority of SEPT1 staining overlapped with that for the Golgi marker GM130, whereas little colocalization was seen for EEA1 or LAMP1, markers of early and late endosomes (Fig. S1D,E). In line with these observations, we found SEPT1 to be enriched in GM130-containing Golgi fractions obtained from HeLa cells (Fig. 1B).

To gain further insight into the sub-compartmental localization of SEPT1 at the Golgi, we used multicolor gated stimulated emission depletion (gSTED) microscopy. SEPT1 staining frequently appeared organized in a stripe-like pattern resembling filaments, aligned along GM130-positive *cis*-Golgi cisternae (Fig. 1C–F). Of note, SEPT1 displayed a non-random distribution with an average distance of individual SEPT1 localizations of 179 nm [Fig. 1E,F; ±112 nm (s.d.) from 944 localizations in ten randomly selected perinuclear areas]. The SEPT1 staining displayed a high degree of correlation with staining for the GM130 and the *cis*-Golgi-associated coat complex COPI (Fig. 1G; Fig. S2A), but was clearly less abundant at the *trans*-Golgi network and at exit sites for cargo proteins, as shown by co-staining with golgin97 (also known as GOLGA1), GGA1 and AP-1 (Fig. 1G; Fig. S2B–D). In addition, SEPT1 was associated with the ER-Golgi-intermediate compartment (ERGIC) (Fig. 1G; Fig. S2E).

Taken together, these results establish the localization and concentration of SEPT1 at early Golgi membranes.

### The structural integrity of the Golgi ribbon depends on SEPT1

**Fig. 2.**
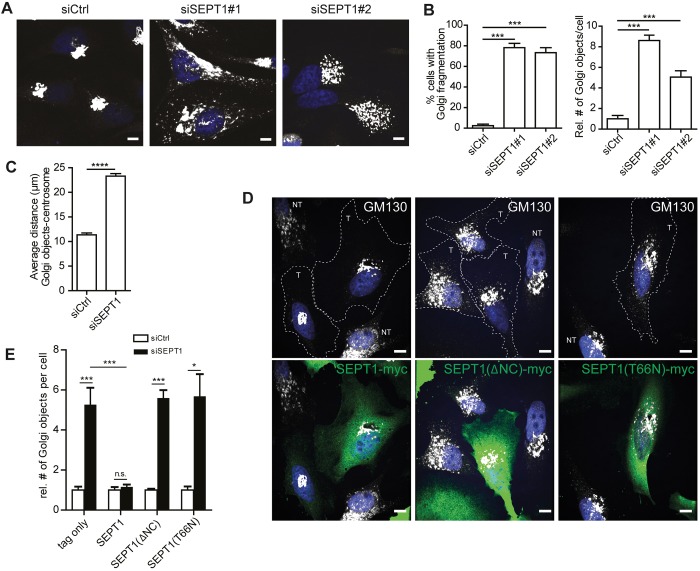
**SEPT1 is required for Golgi integrity.** (A) Immunostaining of a *cis*-Golgi-resident protein (GM130) reveals a significant Golgi fragmentation phenotype in cells depleted of SEPT1. Scale bars: 10 µm. (B) Quantitative analysis of data depicted in A; the proportion of cells displaying a fragmented Golgi, and relative number of Golgi objects per cell in control is shown for siSEPT1#1- or siSEPT1#2-transfected cells (*N*=4; *n*=172–191). ***P<0.001. (C) The average distance between centrosome and Golgi objects increases from 11.37 µm to 23.29 µm upon SEPT1 depletion. Data are represented as mean±s.e.m. ****P<0.0001 (unpaired two-tailed Student's *t*-test; *N*=3; siScr, *n*=98; siSEPT1, *n*=88, t=19.29, d.f.=4). See also Fig. S3F. (D) Myc-tagged, siRNA-resistant SEPT1, SEPT1 (ΔNC) or SEPT1 (T66N) were transfected into siSEPT1#1-treated cells, and Golgi morphology was assessed by immunostaining. T, transfected; NT, non-transfected. (E) Relative number of GM130-positive Golgi objects per cell, quantified from images as depicted in D. Data are represented as mean±s.e.m. *P<0.05; ***P<0.001; ns, not significant (unpaired two-tailed Student's *t*-test). For rescue experiments one-way analysis of variance was performed, followed by a Dunnett post-test, ***P<0.001; *P<0.05; ns, not significant [tag only, *N*=5, *n*=167–191; SEPT1–Myc, *N*=5, *n*=168–184; *P*-value=0.4172; t=0.8554; d.f.=8; SEPT1(ΔNC): *N*=3; *n*=87–110; t=11.55, d.f.=4; SEPT1(T66N): *N*=3; *n*=114-120; t=4.45; d.f.=4].

To gain insight into the function of SEPT1 at the Golgi, we depleted HeLa cells of endogenous SEPT1 by RNAi and monitored the morphology of the Golgi. Both siRNAs efficiently reduced the expression of co-transfected, eGFP-tagged SEPT1 (Fig. S3A), and mRNA levels of endogenous SEPT1 (Fig. S3B). Loss of SEPT1, but not of SEPT2, SEPT6, SEPT7 or SEPT9 (Fig. 2A; Fig. S3C), led to a massive fragmentation of the Golgi, as indicated by an increase in the number of GM130-positive objects. Importantly, no SEPT1-specific immunoreactivity colocalizing with GM130 could be detected in SEPT1-depleted cells (Fig. S3D). Expression of other septin family members remained largely unperturbed upon loss of SEPT1 (Fig. S3E). In many cells, loss of SEPT1 resulted in a scattering of Golgi membranes throughout the cytoplasm (Fig. 2B), as indicated by an increased distance to the centrosome (Fig. 2C; Fig. S3F). This phenotype was specific, as Golgi scattering could be reversed by re-introduction of a variant of SEPT1 into cells that was resistant to siSEPT1#1 (Fig. 2D,E; Fig. S3A,G). Administration of the septin inhibitor forchlorfenuron (FCF), which traps septin monomers in enlarged filaments (Hu et al., 2008), phenocopied what was seen upon loss of SEPT1, but did not trigger its dissociation from Golgi membranes, indicating that SEPT1 needs to be dynamically exchanged at Golgi membranes (Fig. S3H–J).

Filament assembly of septins depends on regions located adjacent to the nucleotide-binding domain (Sirajuddin et al., 2007). We thus generated a SEPT1 mutant lacking N- and C-termini (ΔNC) to assess whether the filament-forming capability of SEPT1 is required to support Golgi integrity. Indeed, unlike wild-type SEPT1 [SEPT1(WT)], this mutant was not incorporated into artificial septin fibers formed upon co-expression with SEPT6–eGFP (Fig. S4A), and could not rescue the disruption to the Golgi integrity seen upon depletion of endogenous SEPT1 (Fig. 2D,E; Fig. S3G), arguing that a filamentous organization of SEPT1 is required for the maintenance of Golgi architecture.

SEPT1 belongs to a subgroup of septins that can hydrolyze GTP, but whether GTP hydrolysis affects the physiological roles of these septins has remained rather enigmatic. Based on sequence comparison we, thus, generated a second mutant of SEPT1 that bears a mutation in threonine 66 (T66N), a residue previously implicated in GTP hydrolysis through the closely related paralog SEPT2 (Sirajuddin et al., 2009). This mutant did not display defects in filament formation when expressed with SEPT6–eGFP (Fig. S4A), but could not rescue the phenotypic defects in Golgi architecture in SEPT1-depleted cells (Fig. 2D,E; Fig. S3G). Thus, SEPT1 needs to be able to hydrolyze GTP for its function at the Golgi. In non-depleted HeLa cells, overexpression of any of the SEPT1 variants (WT, ΔNC or T66N) together with SEPT6–eGFP did not impair Golgi architecture (Fig. S4B).

**Fig. 3.**
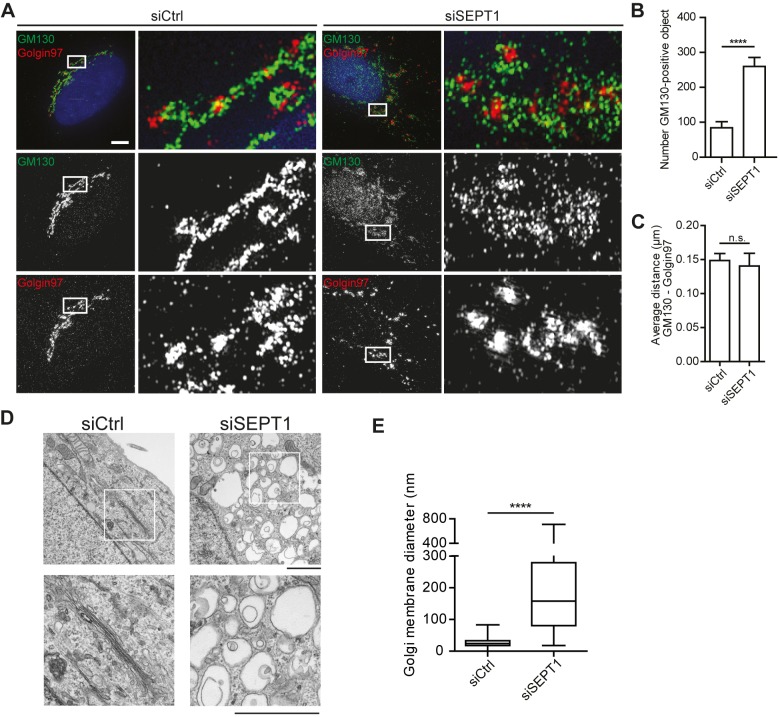
**SEPT1 depletion impairs the ultrastructural organization of the Golgi.** (A) 3D-SIM images of GM130 (green) and golgin97 (red) labeling in HeLa cells after control or SEPT1 depletion. Scale bar: 5 µm. See also Fig. S5 and Movies 1 and 2. (B) Average number of GM130-positive objects observed after control or SEPT1 depletion. Data are represented as mean±s.e.m., unpaired two-tailed *t*-test, ****P<0.0001 (*n*=10; t=5.583; d.f.=18). (C) The average nearest neighbor (NN) distance between GM130-positive objects and the closest Golgin97-positive object remains unaffected upon depletion of SEPT1 (0.149 nm in control and 0.140 in SEPT1-depleted cells). Data are represented as mean±s.e.m., unpaired two-tailed *t*-test, ns, not significant (*P*-value=0.7148). (D) Electron microscopy images derived from control or SEPT1-knockdown cells reveal enlarged vacuole-like structures, instead of Golgi ribbon stacks. Scale bar: 2 µm. (E) The Golgi object diameter increases from 33 to 443 nm upon depletion of SEPT1. Data derived from images as depicted in E (siCtrl: *n*=10; siSEPT1: *n*=13; for each condition 15 cisternae/vacuoles were analyzed per cell). The box represents the 25–75th percentiles, and the median is indicated. The whiskers show the range.

To gain further insight into the structural organization of Golgi fragments formed upon SEPT1 depletion, we made use of structured illumination microscopy (SIM). Our analyses revealed that ERGIC53 (also known as LMAN1)- and GM130-positive objects, demarcating ER-Golgi-intermediate and *cis*-Golgi compartments, respectively, significantly increased in number upon loss of SEPT1 (Fig. 3A,B; Fig. S5A). Despite the overt scattering of the Golgi, *cis*- and *trans*-Golgi compartments remained in close apposition to each other (Fig. 3C; Fig. S5B, Movies 1 and 2). COPI, a coat complex that promotes transport from the Golgi to the ER and between Golgi cisternae, remained associated with the *cis*-Golgi (Fig. S5C). Electron micrographs revealed that, in control cells, the Golgi was organized in stacks of cisternae in the perinuclear area, whereas cells lacking SEPT1 suffered from a massive dilation of presumably early Golgi compartments, as illustrated by a more than 10-fold increase in the average diameter of Golgi objects (Fig. 3D,E). These data demonstrate that SEPT1 stabilizes Golgi cisternae.

Several septin family members have well-established roles during cell division (Fung et al., 2014), and Golgi fragmentation is initiated during mitosis to allow for even distribution between progeny (Persico et al., 2009). Thus, one possible explanation for the fragmentation of the Golgi observed upon loss of SEPT1 might be a defect in Golgi re-assembly during mitosis. If this were true, preventing cells from undergoing mitosis should rescue Golgi fragmentation. We blocked cell cycle progression in G1 by administration of hydroxyurea shortly after transfection of siRNAs; however, this treatment did not rescue the Golgi fragmentation induced by depletion of SEPT1 (Fig. S5D). Also, unlike what is seen upon depletion of other septins (Estey et al., 2010; Spiliotis et al., 2005), loss of SEPT1 did not result in an increase in the number of bi- or multi-nucleated cells (Fig. S5E), indicating that SEPT1-depleted cells exhibit no major defects in cytokinesis. However, SEPT1-depleted cells proliferated at a lower rate compared to control cells (Fig. S5F), consistent with the fact that an intact Golgi is required for cell growth and viability. These data corroborate that Golgi fragmentation in absence of SEPT1 is not caused by defects in cell cycle progression or cytokinesis, but reflects a structural function of SEPT1 at the Golgi.

### SEPT1 is functionally required for release of secretory cargo from the Golgi

**Fig. 4.**
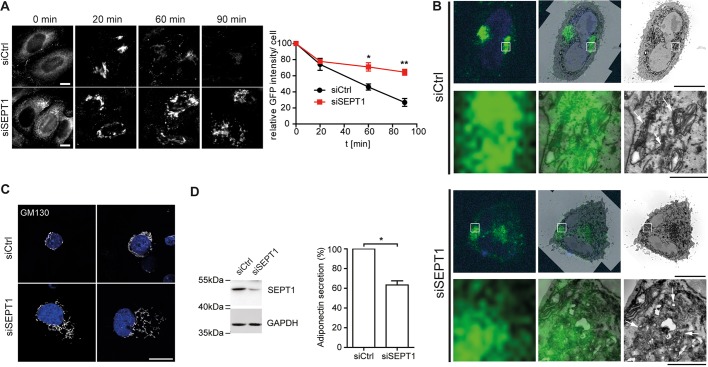
**SEPT1 is required for release of secretory cargo at the Golgi.** (A) Secretion of the FKBP(F36M)–GFP reporter (Gordon et al., 2010) upon induction with D/D solubilizer ligand for the indicated times in control and SEPT1-depleted cells. HeLaM C1 cells were fixed and analyzed by fluorescence microscopy. Scale bar: 10 µm. Time-course of FKBP(F35M)–GFP secretion as quantified by loss of GFP fluorescence over time relative to control cells. Data are represented as mean±s.e.m., unpaired two-tailed Student's *t*-test, *P<0.05, **P<0.01 (*N*=3; 20 min: *n*=277-291; *P*-value=0.6534; t=0.4858; d.f.=4; 60 min: *n*=290-329; t=3.912; d.f.=4; 90 min: *n*=276-307; t=6.175; d.f.=4). See also Fig. S5. (B) Correlative light and electron microscopy analysis of HeLaM C1 cells transfected with siCtrl or siSEPT1#1, and incubated with D/D solubilizer for 20 min to allow for trafficking of the reporter protein to the Golgi. Left panels indicate light microscopy images (green, GFP-tagged reporter protein; blue, nuclei), right panels depict TEM images, and middle panels show overlays. Scale bars: 10 µm for upper panels and 1 µm for bottom panels. (C) Immunostaining of GM130 in control and SEPT1-depleted 3T3 L1 adipocytes. Depicted are two representative cells for each condition. Scale bar: 10 µm. (D) Western blot analysis of adipocyte lysates upon control or SEPT1 depletion (left). Adiponectin secretion from control and SEPT1-depleted adipocytes, as quantified by ELISA (right). Data were normalized to that in control cells (mean±s.e.m.), paired *t*-test, *P<0.05 (*N*=4; t=7.656; d.f.=2).

The Golgi serves as an important sorting station for both anterograde and retrograde membrane traffic. Given the disruption of the Golgi ribbon upon loss of SEPT1, we asked whether SEPT1 might be involved in constitutive secretory membrane traffic. To this end, we made use of a cell line stably transfected with a secretory marker protein (eGFP-tagged human growth hormone fused to multiple copies of the F36M mutant of FKBP) (Gordon et al., 2010), which is only released from the ER and secreted from cells upon addition of a cognate ligand, D/D solubilizer (Rivera et al., 2000). In both control and SEPT1-knockdown cells, the reporter protein reached the Golgi within 20 min of incubation with the solubilizing ligand (Fig. 3A). However, export of the reporter protein from the Golgi, as well as its subsequent release from the cells was markedly delayed upon knockdown of SEPT1 (Fig. 4A; Fig. S5G,H). Based on the observation that ER-to-Golgi transport of the reporter protein remained unperturbed upon SEPT1 depletion, we applied correlative light and electron microscopy to gain insight into the identity of vacuolar structures observed in SEPT1-silenced cells (see Fig. 3D). The localization of the fluorescent reporter protein could be correlated with the Golgi ribbon in the perinuclear area of control cells, as expected (Fig. 4B), whereas in knockdown cells it coincided with scattered and dilated membranes.

To investigate whether loss of SEPT1 also affected secretion of an endogenous cargo, we quantified the release of adiponectin from differentiated 3T3-L1 adipocytes. This adipokine requires proper ER and Golgi function, and leaves the fat cell via endosomal-mediated secretion pathways (Clarke et al., 2006). In 3T3-L1 cells, GM130-positive membranes no longer concentrated in the perinuclear area, the Golgi ribbon was disrupted and the resulting fragments were distributed throughout the cytoplasm in many cells upon SEPT1 knockdown, very similar to the phenotype observed in HeLa cells (Fig. 4C). More importantly, these cells released significantly less adiponectin (Fig. 4D). Taken together, these data strongly indicate that SEPT1 plays an important, and more general role in mediating the exit of secretory cargo from the Golgi.

### SEPT1 requires GM130 for its localization at the Golgi, and recruits centrosomal proteins

**Fig. 5.**
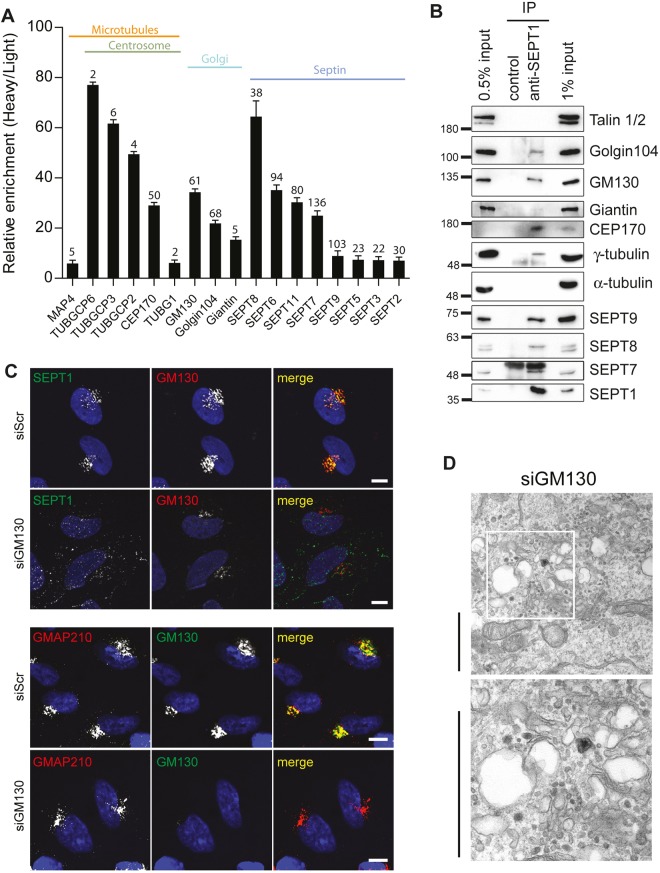
**SEPT1 association with the Golgi requires GM130.** (A) SEPT1-interacting partners identified by mass spectrometry-based screening. For each binding partner ratios of heavy:light (control versus anti-SEPT1 antibody) are plotted. Numbers depicted on top of each bar represent the numbers of peptides attributable to individual proteins found in anti-SEPT1 immunoprecipitates. (B) Endogenous SEPT1 was immunoprecipitated from Jurkat cell lysates, and co-purified proteins were detected by western blot analyses. (C) SEPT1 localizes to the Golgi in a GM130-dependent manner. GM130-depleted and control HeLa cells were fixed with 2% PFA and immunostained for SEPT1 and GM130 (top). Scale bars: 10 µm. Note that loss of GM130 does not perturb the perinuclear concentration of Golgi membranes, as indicated by the localization of GMAP210 (bottom). (D) Electron micrographs derived from GM130-knockdown cells reveal dilated Golgi cisternae in a sub-population of cells (see also Liu et al., 2017), instead of Golgi ribbon stacks (see Fig. 3D for control). Scale bars: 2 µm.

To decipher the molecular determinants underlying SEPT1 localization and function at the Golgi, we performed immunoprecipitation experiments from Jurkat cell lysates, a cell line that expresses high levels of SEPT1, followed by mass-spectrometric analyses to identify co-purified proteins. Individual hits were then validated by immunoblotting, and further probed by immunoprecipitation upon co-expression with SEPT1-myc in HEK293T cells. As expected, several septin family members were found to be enriched in SEPT1 immunoprecipitates (Fig. 5A,B). No staining for SEPT8 was detectable in HeLa cells (data not shown), but, importantly, only SEPT1, but not highly abundant SEPT2, SEPT6, SEPT7 or SEPT9, was prominently enriched at the Golgi (Fig. S6A).

Septin filament assembly can be templated by actin (Kinoshita et al., 2002), and intact actin microfilaments are required to maintain the Golgi ribbon, raising the possibility that a local actin pool might recruit SEPT1 to the Golgi. However, we found no evidence for an association of SEPT1 with actin itself, or with actin-associated and actin-regulatory proteins in our proteomic screen. Also, perturbation of the actin cytoskeleton by means of cytochalasin D or latrunculin B did not release SEPT1 from the Golgi (Fig. S6B).

Instead, we identified several Golgi-resident proteins in SEPT1 immunoprecipitates, including the golgins GM130 and golgin 104 (also known as CCDC186) (Fig. 5A,B). Complex formation between SEPT1 and GM130 or golgin 104 could be validated by immunoprecipitation experiments from transfected Hek293T cells, which revealed a stronger association of SEPT1–Myc with eGFP-tagged GM130 than with eGFP–golgin 104 (Fig. S6C). Accordingly, the recruitment of SEPT1 to Golgi membranes was diminished in cells depleted of GM130 (Fig. 5C) (Lee et al., 2014), but not in cells lacking expression of golgin 104 (data not shown), demonstrating that GM130 is required to anchor SEPT1 at the Golgi. In support of a functional interplay between SEPT1 and GM130, cells derived from GM130-knockout animals have been reported to display a fragmented Golgi (Liu et al., 2017), a feature that also became apparent in a sub-population of GM130-depleted HeLa cells (Fig. 5D).

**Fig. 6.**
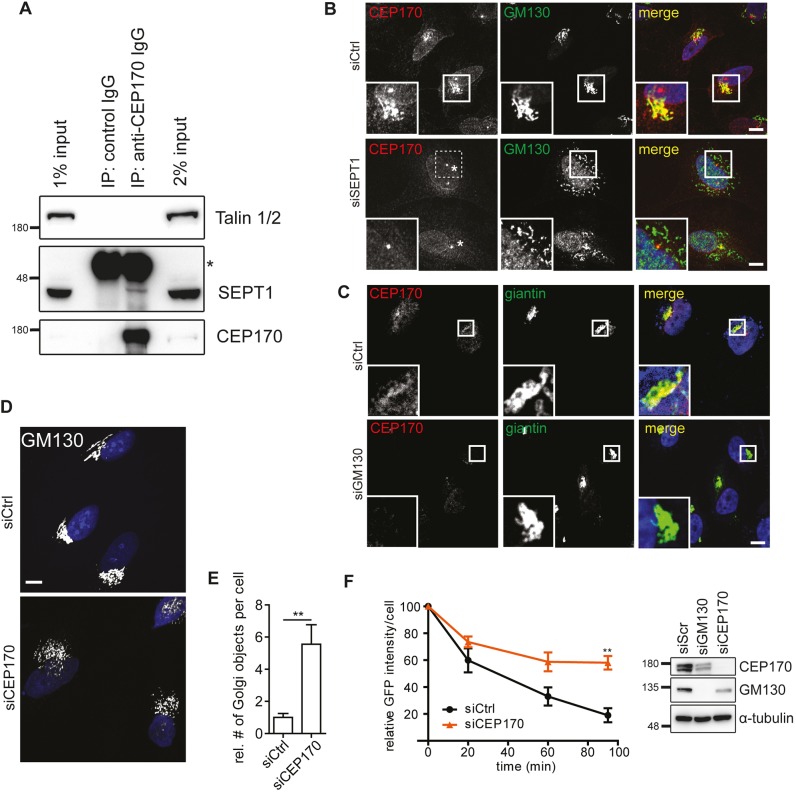
**Golgi-associated SEPT1 connects to microtubule-nucleating factors.** (A) Endogenous SEPT1 co-purifies with CEP170 from Jurkat cell lysates (asterisk indicates heavy chains of antibodies used for immunoprecipitation). (B) CEP170 localization to early Golgi membranes requires SEPT1 (for centrosomal localization of CEP170, see also Fig. S7C). Control or SEPT1-depleted HeLa cells were fixed with 2% PFA and immunolabeled for CEP170 and GM130. Scale bars: 10 µm. Note that in siSEPT1-treated cells the localization of CEP170 to the centrosome remains unperturbed (indicated by the asterisk). (C) GM130 is required for the localization of CEP170 at giantin-positive Golgi membranes. Scale bar: 10 µm. (D) Depletion of CEP170 induces Golgi fragmentation. Individual Golgi objects were visualized by immunolabeling for GM130. Scale bar: 10 µm. (E) Relative number of Golgi objects in CEP170-depleted cells. Data are represented as mean±s.e.m., unpaired two-tailed Student's *t*-test, **P<0.01 (*N*=5; *n*=112–195; t=3.67; d.f.=8). (F) FKBP(F35M)–GFP secretion in control, or CEP170-depleted cells, represented as mean±s.e.m., one-way analysis of variance, followed by a Dunnett post-test, **P<0.01 (siScr: *N*=6; *n*=417; siCEP170: *N*=3; *n*=75) (left). Western blot analysis of HeLa cell lysates upon control (siCtrl), GM130 or CEP170 depletion (right). Note that expression levels of GM130 and CEP170 appear to be correlated.

To our surprise, several centrosomal proteins were co-purified with SEPT1, including CEP170 and components of γ-Turc, such as γ-tubulin and GCP2, GCP3 and GCP6 (also known as TUBGCP2, TUBGCP3 and TUBGCP6, respectively) (Fig. 5A,B), whereas α-tubulin was not detectable (Fig. 5B). Immunoprecipitation experiments from transfected HEK293T cells corroborated the association of Myc-tagged SEPT1 with full-length eGFP-tagged CEP170, whereas a fragment comprising the microtubule-binding C-terminal domain of CEP170 displayed a much weaker association. This indicates that SEPT1 binding to CEP170 does not interfere with its association with microtubules (Fig. S7A). Conclusively, endogenous SEPT1 could be co-purified with CEP170 from cell lysates (Fig. 6A). Likewise, Myc-tagged SEPT1 was found to associate with mCherry-tagged γ-tubulin (Fig. S7B), indicating that SEPT1, CEP170 and γ-tubulin form a complex.

Given its interaction with SEPT1, we next studied the subcellular distribution of CEP170. In methanol-fixed cells, endogenous CEP170 could be detected at the centrosome, as previously described (Guarguaglini et al., 2005), where it colocalized with pericentrin (Fig. S7C). An additional pool became apparent at the Golgi upon fixation with paraformaldehyde (PFA), a condition that does not perturb the integrity of septin filaments. Of note, this pool colocalized with GM130 (Fig. 6B), and could no longer be detected in cells depleted of SEPT1 or GM130 (Fig. 6B,C). The localization of CEP170 at the centrosome was unperturbed in cells lacking SEPT1 expression. This demonstrates that GM130-associated SEPT1 mediates the recruitment of CEP170 to the Golgi. Furthermore, depletion of CEP170 resulted in Golgi fragmentation (Fig. 6D,E) (Guarguaglini et al., 2005), and impaired secretion to a similar extent to that seen upon depletion of SEPT1 (Fig. 6F, Fig. S7D), indicating that SEPT1 and CEP170 have overlapping functions. Conversely, overexpressed CEP170 decorated microtubules (Guarguaglini et al., 2005), and triggered the redistribution of a sub-population of SEPT1 to microtubules (Fig. S7E).

These findings identify a SEPT1-based scaffold that physically and functionally links the Golgi to microtubule-organizing factors, such as CEP170 and γ-tubulin.

### The SEPT1 scaffold facilitates the nucleation of microtubules at the Golgi

**Fig. 7.**
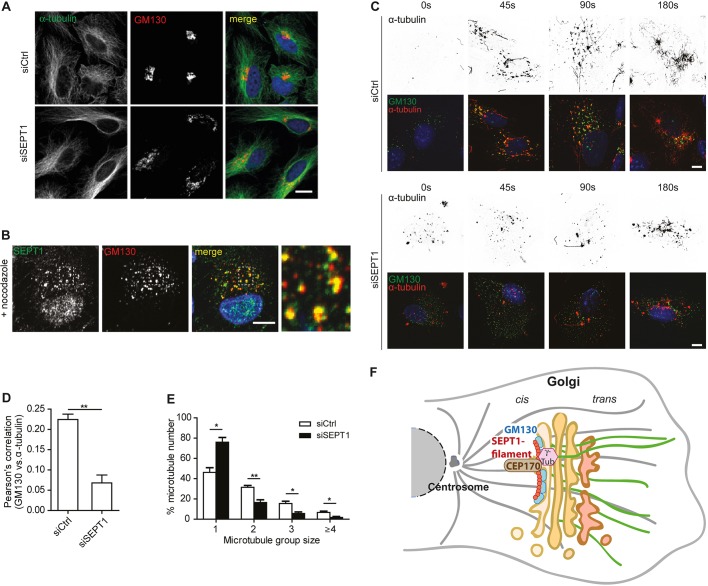
**SEPT1 facilitates microtubule nucleation at the Golgi.** (A) Staining of α-tubulin and GM130 in control and in SEPT1-depleted cells. (B) SEPT1 localization to GM130-positive Golgi objects in RPE1 cells is not affected by nocodazole administration. Scale bar: 10 µm. (C) GM130- and α-tubulin staining in control and SEPT1-depleted RPE1 cells, fixed at the indicated time points after nocodazole washout. Scale bar: 10 µm. (D) The Pearson's correlation coefficient of α-tubulin and GM130 staining decreases upon depletion of SEPT1, demonstrating that α-tubulin becomes less abundant at early Golgi membranes. Data are represented as mean±s.e.m., unpaired two-tailed Student's *t*-test, **P<0.01 (*N*=3; siScr: *n*=176; siSEPT1: *n*=96; t=6.613; d.f.=4). (E) Number of microtubules nucleated in 45 s upon nocodazole washout as determined for individual GM130-positive objects in control and SEPT1-depleted RPE1 cells (see also Fig. S7H). Data are represented as mean±s.e.m., unpaired two-tailed Student's *t*-test, **P<0.01, *P<0.05, (microtubule group size 1: t=4.478; d.f.=4; microtubule group size 2: t=4.652; d.f.=4; microtubule group size 3: t=3.65; d.f.=4; microtubule group size ≥4: t=3.085; d.f.=4). (F) Model for SEPT1 function at the Golgi. GM130 (in blue) recruits SEPT1 (in red) to *cis*-Golgi membranes. SEPT1 is organized in a filament-like fashion and serves as an anchor for the association of CEP170 (brown) and γ-tubulin (pink) to nucleate Golgi-derived microtubules (green).

Given the association of SEPT1 with microtubule-organizing factors, we first asked whether SEPT1 depletion perturbed microtubule organization. However, at the steady-state no striking differences could be detected between control and SEPT1-knockdown cells (Fig. 7A), also with regard to post-translational modifications of tubulin (acetylation or tyrosination, data not shown). We next addressed the question of whether an intact microtubule cytoskeleton was required for the localization of SEPT1 at the Golgi. Nocodazole treatment triggered the fragmentation of the Golgi, as expected, and this was reversed by nocodazole washout (Fig. 7B). However, SEPT1 remained associated with Golgi fragments induced by nocodazole application, demonstrating that the association of SEPT1 with the Golgi is independent of the microtubule network. Interestingly, shortly after washout, we found SEPT1 frequently to be localized between neighboring GM130-positive objects (Fig. S7F).

Based on the association of SEPT1 with CEP170 at *cis*-Golgi membranes, and the fact that we could detect endogenous γ-tubulin in CEP170 immunoprecipitates from Jurkat cell lysates (Fig. S7G), we speculated that SEPT1 was involved in microtubule nucleation at the Golgi. Indeed, staining of α-tubulin at different time points after nocodazole washout revealed a marked delay in microtubule re-growth in SEPT1-depleted cells (Fig. 7C).

A specific pool of microtubules is nucleated at the Golgi in many cell types (Sanders and Kaverina, 2015). Based on the ability of SEPT1 to physically link the Golgi to centrosomal proteins, we thus hypothesized that the SEPT1 scaffold may be specifically involved in this process. Indeed, in control cells, a subset of GM130-positive objects was decorated with α-tubulin 90 s after nocodazole washout in control cells, whereas significantly less α-tubulin was associated with *cis*-Golgi membranes in SEPT1-knockdown cells (Fig. 7D). Furthermore, individual GM130-positive objects nucleated a markedly reduced number of microtubules (Fig. 7E; Fig. S7H). These data, thus, demonstrate that SEPT1 facilitates the nucleation of Golgi-derived microtubules.

## DISCUSSION

Our study has revealed a novel and surprising role for septins in maintaining Golgi architecture, positioning and function. SEPT1 resides predominantly at early Golgi compartments, and promotes the association of centrosomal proteins, including CEP170. SEPT1 thereby facilitates the nucleation of Golgi-derived microtubules. As a consequence, depletion of SEPT1 triggers a massive fragmentation of the Golgi ribbon, causes a dilation of Golgi cisternae and strongly impairs anterograde and retrograde membrane trafficking events (Fig. 7F).

### SEPT1 functions independently of other septin family members

Septins are generally believed to act as hetero-oligomeric filaments, raising the question of whether SEPT1 function at the Golgi depends on other septin family members. Indeed, our super-resolution images revealed a non-random distribution of SEPT1 at the Golgi, and the staining pattern suggests a filamentous organization. However, we were unable to find any other, highly abundant septin family member, such as SEPT2, SEPT6, SEPT7 or SEPT9, as prominently enriched at the Golgi as SEPT1. More importantly, depletion of none of these paralogs resulted in Golgi fragmentation, although non-redundant SEPT7 and SEPT9 are required for the assembly of stable heteromeric filaments (Sellin et al., 2011). As a SEPT1 mutant deficient in stable filament assembly could not rescue the Golgi fragmentation induced by SEPT1 depletion (Fig. 2D,E; Fig. S3G), our data indicate that SEPT1 at the *cis*-Golgi functions as a homodimer, or as a homo-oligomeric multimer. However, how the regular distribution of SEPT1 at the Golgi is established, remains yet to be clarified.

Interestingly, we found SEPT1 to link individual Golgi objects upon nocodazole washout at a stage when ministacks are re-connected to form an intact Golgi ribbon (Fig. S7F). Oligomerization of Golgi-associated SEPT1 into filaments at this point may, thus, support the formation of elongated cisternae. This could also explain why Golgi cisternae are dilated in absence of SEPT1 (Fig. 3D). Furthermore, we show that treatment with FCF, a drug that dampens turnover of septin monomers in filaments, triggered Golgi fragmentation (Fig. S3H–J) without impairing SEPT1 recruitment to Golgi objects, indicating that SEPT1 filaments need to undergo dynamic rearrangements to support Golgi maintenance. Septin filaments can associate laterally with each other through their C-terminal coiled-coil domains (Estey et al., 2011), and thereby form higher ordered bundles. Golgi-associated SEPT1 could, therefore, also facilitate stacking of neighboring cisternae. However, it is important to note that SEPT1 is apparently not essential for membrane adhesion between neighboring cisternae in general, as *cis*- and *trans*-Golgi subcompartments remain juxtaposed, irrespective of the presence of SEPT1 (Fig. 3A,C).

### SEPT1 association with *cis*-Golgi membranes requires GM130

The integrity of the Golgi ribbon depends on actin microfilaments (Egea et al., 2013), and actin filaments can serve as a template for septin assembly (Kinoshita et al., 2002). However, we found no evidence for an association of SEPT1 with actin or actin regulatory proteins, suggesting that actin is not involved in the recruitment of SEPT1 to the Golgi. In line with this, neither cytochalasin D nor latrunculin B treatment released SEPT1 from *cis*-Golgi compartments.

Instead, the association of SEPT1 with early Golgi compartments depends on GM130, a golgin that engages in binding to a plethora of factors involved in ribbon assembly, vesicle tethering and fusion, microtubule nucleation and centrosome organization (Nakamura, 2010). Thus, depletion of GM130 is expected to profoundly alter the protein composition of Golgi membranes, and might also indirectly affect the recruitment of SEPT1. However, it is important to note that loss of GM130 phenocopies most of the defects observed upon SEPT1 depletion, including alterations in Golgi positioning (Liu et al., 2017), a dilation of Golgi cisternae (Fig. 5D), the release of CEP170 from the Golgi (Fig. 6B) and impaired microtubule nucleation at the Golgi (Rivero et al., 2009). In addition, secretion is impaired in cells lacking GM130 expression (K.S., unpublished observations, and Liu et al., 2017), further substantiating a functional association of SEPT1 with GM130. Future studies will need to address the question of whether SEPT1 takes over modulatory functions on interactions between GM130 and its binding partners.

### SEPT1 aids the nucleation of Golgi-derived microtubules

Several septins have been implicated in the regulation of microtubule stability, either by directly associating with microtubules (Bai et al., 2013), or by modulating the activities of microtubule-associated proteins, like MAP4 (Kremer et al., 2005; Spiliotis et al., 2008), or of microtubule-modifying enzymes (Ageta-Ishihara et al., 2013). SEPT1, however, does not align with microtubules, but rather facilitates their nucleation at Golgi membranes (Fig. 7F), highlighting a distinct function for this family member. We reveal that SEPT1 associates with a number of γ-Turc-associated proteins and with CEP170. Though not involved in the nucleation of microtubules at the centrosome (Guarguaglini et al., 2005), CEP170 has recently been shown to stimulate microtubule nucleation at aberrant clusters formed upon overexpression of a fragment of AKAP450 (Kolobova et al., 2017), and at the surface of the inclusion membrane surrounding intracellular pathogens (Dumoux et al., 2015). Based on our observations we propose that CEP170 is, thus, more specifically required for connecting the SEPT1/GM130 scaffold to the microtubule-nucleating machinery at the Golgi ribbon. Indeed, depletion of SEPT1 releases CEP170 from the Golgi without affecting its recruitment to the centrosome (Fig. 6B). We found that both CEP170 and SEPT1 associated with γ-tubulin (Figs 5B and 6A), and could further demonstrate that cells depleted of CEP170 display similar defects in Golgi architecture (Fig. 6D,E) and secretion (Fig. 6F; Fig. S7D) when compared to SEPT1-knockdown cells. It remains to be determined whether and how the GM130–SEPT1–CEP170 scaffold identified in this study integrates additional factors, such as MAP4 (Kremer et al., 2005; Spiliotis et al., 2008), AKAP450 (Rivero et al., 2009) or CLASP proteins (Efimov et al., 2007), to further support microtubule nucleation, or to stabilize newly formed microtubules.

Interestingly, CEP170 is redistributed to spindle microtubules during mitosis, presumably triggered by phosphorylation through Polo-like kinase 1 (Guarguaglini et al., 2005). As we observed that overexpressed CEP170 recruits endogenous SEPT1 to microtubules (Fig. S7E), this indicates that the SEPT1–CEP170 scaffold might fulfill alternative functions during cell cycle progression. In line with this possibility, overexpressed SEPT1 has been show to be associated with spindle poles and the nearby microtubule region during cytokinesis, and is a known target of Aurora B kinase (Qi et al., 2005). Future studies need to elucidate the functional interplay between CEP170 and endogenous SEPT1 at this stage of the cell cycle, and to understand why loss of SEPT1 delays cell cycle progression (Fig. S5F).

### Implication of the SEPT1-based scaffold for exit of secretory cargo at the Golgi

Consistent with established functions of Golgi-derived microtubules (Sanders and Kaverina, 2015), we could demonstrate that depletion of SEPT1 or CEP170 profoundly impairs secretion (Figs 4A,D and 6F). Importantly, transport of a reporter protein from the ER to the Golgi remained unperturbed in cells lacking expression of SEPT1, indicating that SEPT1 was not involved in membrane traffic between these two organelles. We conclude that SEPT1 has a predominant role in release of cargo from the Golgi, a function that is strikingly different to established roles of other septin family members that impact on secretion at post-Golgi steps of exocytosis (Beites et al., 2005; Beites et al., 1999; Tokhtaeva et al., 2015). This function in secretory transport is not restricted to HeLa cells, as we could demonstrate that loss of SEPT1 also impedes secretion of the fat cell-derived hormone adiponectin in differentiated adipocytes (Fig. 4D), pointing towards a role of SEPT1 in the crosstalk between adipose tissue and other key metabolic tissues, and, thus, also in whole body energy homeostasis (Wang and Scherer, 2016). It will be interesting to see whether and how SEPT1 is involved in the release of further cargoes in other secretory cells.

## MATERIALS AND METHODS

### Cell lines and cell culture

All cell lines used for this study were tested for contaminations on a regular basis. HeLa (ATCC), HeLaM C1 (Gordon et al., 2010), Cos-7 (ATCC) and HEK293T (ATCC) cells were cultured in Dulbecco's modified Eagle's medium (DMEM) with 4.5 g l^−1^ glucose, 10% heat-inactivated fetal bovine serum (FBS) and 50 µg ml^−1^ penicillin and streptomycin. Retinal pigmented epithelial (RPE-1, ATCC) cells were cultured in DMEM supplemented with nutrient mixture F-12, GlutaMAX^TM^-I, 10% FBS and 50 µg ml^−1^ penicillin and streptomycin. Jurkat cells (ATCC) were cultured in Roswell Park Memorial Institute (RPMI) 1640 medium with 10% FBS and 50 µg ml^−1^ penicillin and streptomycin.

3T3-L1 fibroblasts (ATCC) were cultured in Iscove's modified Dulbecco's medium (IMDM, 4.5 g l^−1^ glucose) with 10% newborn calf serum (NCS) at 37°C and were differentiated in IMDM containing 10% FBS, 1.2 µg ml^−1^ insulin, 0.5 mM 3-isobutyl-1-methylxanthine (IBMX), 0.25 µM dexamethasone and 2 µM rosiglitazone as soon as they had reached confluence. At 2 or 3 days after differentiation, medium was changed to fresh IMDM containing 10% FBS and 1.2 µg ml^−1^ insulin.

### Antibodies and plasmids

An antibody recognizing SEPT1 was derived from rabbits immunized with the peptide CRASRSKLSRQSATEI coupled to KLH (Eurogentec), followed by affinity-purification on GST-tagged SEPT1 cross-linked on glutathione-coupled beads, or on the peptide cross-linked to a matrix. The antibody recognizing SEPT9 has been described previously (Diesenberg et al., 2015). An antibody detecting COPI was kindly provided by the Felix Wieland (Heidelberg University Biochemistry Center, Germany) (Faulstich et al., 1996). Commercial antibodies recognizing SEPT1 (sc-398586), SEPT7 (sc-20620), ERGIC53 (sc-398893) or Mmyc (clone 9E-10) were from Santa Cruz Biotechnology. Antibodies detecting SEPT2 (HPA018481), SEPT6 (HPA005665), α-tubulin (T5168), GAPDH (G8795), golgin104 (HPA018019) or talin (T3287) were obtained from Sigma-Aldrich. Antibodies against GM130 (610822), EEA1 (610456), AP-1 (610386), dynactin (610473), GMAP210 (611712) or LAMP1 (555798) were purchased from BD Bioscience. Antibodies raised against γ-tubulin (ab11316), GM130 (ab52649), Vps26 (ab23892), CEP170 (ab72505) or TGN46 (ab50595) were from Abcam. Antibodies detecting SEPT9 (H00010801) or GGA1 (H00026088-B01) were purchased from Abnova. Antibodies specific for CEP170 (41-3220) and golgin97 (A-21270) were obtained from Invitrogen. GFP was detected with an antibody from Clontech (632381), giantin with an antibody from Biolegend (924302). Antibody dilutions used in this study to detect individual proteins by western blotting or by indirect immunofluorescence are listed in Table S1.

All SEPT1 plasmids for mammalian cell expression were based on a human cDNA clone (accession number BC012161). Tagged or truncated variants were generated by sub-cloning of SEPT1 into a pcDNA3.1-based vector, leading to fusion of eGFP- or Myc-encoding sequences to the 3′-end of SEPT1. To generate the GTP hydrolysis-deficient mutant T66N, the primer 5′-TTGACACAGAaCCTGGCCATTGA-3′ was used for site-directed mutagenesis (the mutation is indicated by the lowercase letter). The ΔNC-mutant of SEPT1 comprised amino acids 23–305 of full-length SEPT1. Variants of SEPT1 resistant to siSEPT1#1 were generated by site-directed mutagenesis using the primer 3′-GGAAGGAGGAAGAAATTCACATCTAC-5′.

The cDNAs encoding rat GM130 (a gift from Nobuhiro Nakamura, Kyoto Sangyo University, Japan) (Nakamura et al., 1995) and human Golgin-104 (accession number BC105632) were fused to the 3′-end of eGFP by subcloning into a pcDNA3.1-based vector. pEGFP-CEP170 (Nigg PID200) and pEGFP-CEP170-Cterm (Nigg PID GG2) were from Addgene (deposited by Erich Nigg; plasmids #41150 and #41151) (Guarguaglini et al., 2005). mCherry-γ-tubulin-17 was a gift from Michael Davidson (Addgene plasmid #55050).

### Transient transfection

Small-interfering RNAs targeting human gene expression had the following sequences (5′–3′): siCtrl, AUCGUUGACUUACAAGAGAdTdT; siSEPT1#1, GGAAGAGGAGAUCCACAUCdTdT; siSEPT1#2, GCAGGAAAGUGGAGAAUGAdTdT; siSEPT2, GGAUGAAAUUGAAGAACAUdTdT; siSEPT6, AGAUCCGAAGAGUGCUACAdTdT; siSEPT7, CUUGCAGCUGUGACUUAUAdTdT; siSEPT9, GGAGGAGGUCAACAUCAACdTdT (Diesenberg et al., 2015); siGM130, GGACAAUGCUGCUACUCUACAACCAdTdT (Lee et al., 2014); and siCEP170, GAAGGAAUCCUCCAAGUCAdTdT (Guarguaglini et al., 2005).

siRNAs were transfected into cells, and cells were cultured for 48–56 h before further experiments. For treatments with siSEPT1#2 and with siGM130, cells were split 36 h after the initial transfection, and re-transfected with siRNA once more 12 h later. siSEPT1#1 was transfected for all those experiments, in which effects of SEPT1 were probed with a single SEPT1-targeting siRNA.

DNA and siRNA transfections were performed with JetPRIME (Polyplus transfection) and Oligofectamine (Invitrogen), respectively, according to the manufacturer's instructions. HEK293-T cells were transfected using the calcium phosphate method. DNA was mixed with 0.25 M CaCl_2_ and incubated for 5 min at room temperature. The same volume of 2× HBSS (280 mM NaCl, 0.05 M HEPES, 1.5 mM Na_2_HPO_4_, pH 7.0) was added while stirring. After 20 min of incubation at room temperature, the solution was added to the cells.

Expression of SEPT1 in mouse cells was silenced by transfecting a smart pool obtained from Dharmacon (M-045599-01-0050); in this case UAAGGCUAUGAAGAGAUACUU was used as a non-targeting control siRNA. Mature 3T3-L1 cells were transfected 5 days after differentiation by electroporation in an electroporation cuvette (4 nm electrode gap) containing siRNA (6 nmol per six-well plate) at 170 V and 960 µF with the Gene Pulser Xcell^TM^ (Bio-Rad Laboratories). Cells were cultured for 96 h before further experiments.

### RT-PCR

Cells were transfected with siCtrl, siSEPT1#1 or siSEPT1#2, and harvested 48 h later. RNA was purified from cell pellets using the RNeasy Plus mini kit (Quiagen), and cDNA was prepared from 400 ng of RNA using the SuperScript IV kit (ThermoFisher scientific). PCR reactions were performed in technical duplicates using the primer pairs 5′-CATGTACGTTGCTATCCAGGC-3′/5′-CTCCTTAATGTCACGCACGAT-3′ and 5′-CAGAAGATCCGGGATCAGTT-3′/5′-CTCCACCTCCACGGTC-3′ to amplify actin B and SEPT1 cDNAs. PCR products were separated by agarose gel electrophoresis, and band intensities were quantified by densitometry. Values obtained for SEPT1 were normalized to actin levels detected in the same samples.

### Isolation of a Golgi-enriched fraction

HeLa cells were trypsinized and harvested by centrifugation at 500 ***g*** for 10 min. Pelleted cells were washed twice with ice-cold PBS and once with ice-cold homogenate buffer (250 mM sucrose, 10 mM Tris-HCl pH 7.4) and resuspended with homogenate buffer to have a final volume equal to five times the volume of the cell pellet. Resuspended cells were homogenized with a Balch homogenizer (gap size 12 µm) with 20 strokes at 4°C. Cell homogenate was centrifuged at 600 ***g*** for 10 min at 4°C, and the supernatant was mixed with 62% (w/w) sucrose solution and EDTA (pH 7.1) to obtain a homogenate with 37% (w/w) sucrose and 1 mM EDTA. 4 ml of homogenate were transferred into a SW40 tube (Beckman) and overlaid with 5 ml of 35% (w/w) sucrose solution in 10 mM Tris-HCl (pH 7.4), and 4 ml of 29% (w/w) sucrose solution in 10 mM Tris-HCl (pH 7.4). The gradient was centrifuged at 100,000 ***g*** for 160 min at 4°C, and the Golgi-enriched fraction was collected with a syringe (22G needle) at the interface between the 35% and 29% sucrose layers. Four volumes of PBS were added to one volume of fraction and centrifuged at 100,000 ***g*** for 30 min at 4°C. Pelleted Golgi membranes were resuspended with Laemmli buffer and further analyzed by western blotting [protocol adapted from Kaloyanova et al. (2015)].

### Immunoprecipitation

Jurkat or transfected HEK293T cells were harvested by centrifugation (400 ***g*** for 5 min), and the cell pellet was washed once in PBS. Cells were resuspended in buffer A (20 mM HEPES pH 7.4, 100 mM KCl, 2 mM MgCl_2_, 1% Triton-X-100) and incubated on ice for 20 min. The resulting lysate was centrifuged at 17,000 ***g*** for 20 min at 4°C. 1 ml of supernatant (containing 2–4 mg of protein for HEK293T- and 5–10 mg of protein for Jurkat-derived cell lysates, respectively) was then added to protein A/G–agarose coupled to appropriate primary antibodies or to Nano-Traps (ChromoTek) retaining eGFP- or mCherry-tagged proteins, and incubated with end-over-end rotation for 2–3 h at 4°C. For immunoprecipitation experiments from Jurkat cells, highly cross-absorbed goat-anti-rabbit-IgG antibodies were used as controls. Beads were then washed four times in buffer A, and once in buffer A lacking detergent. Retained material was then eluted in Laemmli buffer and analyzed by mass spectrometry (as detailed in Mössinger et al., 2007).

### Immunofluorescence

Cells were fixed in 2% PFA, in 4% PFA or in methanol, and washed twice in 120 mM Na_x_H_x_PO_4_, pH 7.4, and twice in high-salt PBS (0.1% Triton X-100, 150 mM NaCl and 3.3 mM Na_x_H_x_PO_4_, pH 7.4 in PBS). After blocking in goat serum dilution buffer (GSDB, 3.3% goat serum, 150 mM NaCl, 6.6 mM Na_x_H_x_PO_4_ and 0.1% Triton X-100 in PBS) for 20 min, primary antibodies diluted in GSDB were incubated with cells for 1 h at room temperature. Then, excessive primary antibodies were washed away three times in high-salt PBS for 10 min, and Alexa-Fluor-coupled, secondary antibodies diluted in GSDB were incubated with cells for 1 h at room temperature. Prior to mounting, cells were washed twice in high-salt PBS for 5 min and twice in 120 mM Na_x_H_x_PO_4_ for 5 min.

### Secretion assay

A HeLaM cell line stably expressing an eGFP-tagged FKBP reporter construct (C1) [kindly provided by Andrew Peden, University of Sheffield, UK (Gordon et al., 2010)] was used to monitor constitutive secretion. The reporter protein contains a series of mutant FKBP moieties (F36M), which form large aggregates that stay in the ER, but these aggregates are solubilized and secreted into the medium upon addition of a ligand (D/D Solubilizer, Clontech). Control and knockdown cells were fixed with 4% PFA at different time points after ligand treatment, and their secretory capacity was analyzed by immunofluorescence microscopy.

### Adiponectin secretion assay

To determine the level of secreted adiponectin in control or SEPT1-knockdown 3T3-L1 adipocytes, cells were washed with PBS, and 500 µl of serum-free IMDM containing 100 nM insulin and 1% penicillin/streptomycin were added per well in a 12-well plate. 3T3-L1 adipocytes were incubated for 24 h at 37°C. Then the medium was recovered and the remaining material was centrifuged for 5 min at 500 ***g*** at room temperature to remove cell debris. Adiponectin levels in the collected media were measured with an enzyme-linked immunosorbent assay (ELISA) (DY1119, R&D Systems GmbH) following the manufacturer's instructions. Cells were washed with PBS and lysed to determine protein concentration. For quantification, adiponectin levels were normalized to the respective protein concentration.

### Microtubule nucleation assay

The nucleation of microtubules was measured in RPE-1 cells essentially as described previously (Efimov et al., 2007). Briefly, cells were incubated in medium containing 2.5 µg ml^−1^ of nocodazole for 2 h in order to depolymerize microtubules. Nocodazole was then washed out by rising cells with ice-cold medium five times, and microtubule regrowth was induced by adding warm medium. Warm medium were removed at different time points (0, 45, 90 and 180 s), and cells were incubated in extraction buffer (60 mM PIPES, 25 mM HEPES, 10 mM EGTA, 2 mM MgCl_2_, 0.1% saponin, 0.25 nM nocodazole, and 0.25 nM taxol, pH 6.9) for 40 s prior to fixation.

### Confocal spinning-disc microscopy

All fluorescence images were obtained by confocal spinning-disc microscopy, unless indicated otherwise, using an Ultra View ERS Rapid Confocal Imager (Perkin Elmer), equipped with an EM-CCD camera (Hamamatsu) and a Zeiss Axiovert 200 M microscope controlled by Volocity software (Perkin Elmer). The same software was used for image processing and quantifications.

### Gated stimulated emission depletion microscopy

gSTED imaging with time-gated detection was performed on a Leica SP8 TCS STED microscope (Leica Microsystems) equipped with a pulsed white light excitation laser (NKT Photonics). Dual-channel STED imaging was performed by sequentially exciting ATTO647N and Alexa Fluor 594 at 646 nm and 598 nm, respectively.

Both dyes were depleted with a 775 nm STED laser. A single optical section was acquired with an HC PL APO CS2 100×/1.40 NA oil objective (Leica Microsystems), a scanning format of 1024×1024 pixel, 8-bit sampling, and 6-fold zoom, yielding a pixel dimension of 18.9×18.9 nm. Time-gated detection was set from 0.3–6 ns for all dyes. Fluorescence signals were detected sequentially by hybrid detectors at the appropriate spectral regions separated from the STED laser. To minimize thermal drift, the microscope was housed in a heatable incubation chamber (LIS Life Imaging Services).

The effective lateral resolution of the SP8 TCS STED microscope was previously determined using 40 nm fluorescent beads (Life Technologies; excitation/emission maxima at 505/515 nm or 660/680 nm) to be 45 nm for the 647 nm channel (Grauel et al., 2016). Raw data obtained from gSTED imaging were analyzed for colocalization using Pearson correlation (Coloc 2, ImageJ) from 9–21 randomly selected cells. Nearest neighbor distances of the SEPT1 gSTED images were calculated from 10 randomly selected regions in the Golgi or nuclear area after auto-thresholding (Bernsen, ImageJ) and size selection of >5 pixel² using Nearest Neighbour Distances Calculation (ImageJ; https://icme.hpc.msstate.edu/mediawiki/index.php/Nearest_Neighbor_Distances_Calculation_with_ImageJ).

### Structured-illumination microscopy

Samples were mounted in Vectashield. 3D 3-color SIM images were acquired using the 488 nm, 568 nm and 643 nm laser lines, standard filter sets and 125 nm *z*-sectioning of the OMX V4 Blaze (GE Healthcare) system. 100 nm fluorescent beads (Tetraspeck, T7284, Thermo Fisher Scientific) were used for registration of detection channels achieving less than 40 nm registration error for all three channels.

3D-rendering and image export was performed with Arivis Vision4D. Objects were identified with a histogram-based threshold procedure (Otsu's method) and holes of up to one voxel (0.02 µm³) were filled. Average nearest-neighbor distances were calculated from boundaries of rendered 3D objects, using a custom-written Python script (available from the corresponding author upon request). Size and number of objects were calculated with ImageJ (Schneider et al., 2012) after histogram-based thresholding (Otsu's method).

### Electron microscopy

Samples were fixed with 2.5% glutaraldehyde in PBS for 30 min, then washed and fixed again with 1% osmium tetroxide and 1.5% potassium hexacyanoferrate. Then, the samples were dehydrated in a methanol gradient and propylene oxide, and flat embedded in epoxy resin. Coverslips were removed with liquid nitrogen after polymerization. Cells were ultrathin-sectioned, and the Golgi compartment was analyzed using a Zeiss 900 transmission electron microscope. Images were processed and quantified with Image J.

#### Correlative light and electron microscopy

HeLaM C1 cells stably expressing an eGFP-tagged FKBP reporter construct (Gordon et al., 2010) were transfected with siCtrl or siSEPT1#1 as described above, and then split onto Matrigel-coated coverslips with an etched numbered grid. The expression of the reporter protein was induced by addition of D/D solubilizer, and cells were fixed for 45 min in PFA. After staining of nuclei, cells were imaged by confocal spinning disk microscopy in 3D. Afterwards, cells were post-fixed in 2% glutaraldehyde in PBS, washed and processed for Durcupan embedding (Deerinck et al., 2018). Cells were incubated in 2% OsO_4_/1.5% C_6_N_6_FeK_4_ in PBS for 1 h at room temperature, washed and transferred into 1% aqueos thiocarbohydrazide for 20 min. Cells were washed, incubated in 2% aqueous OsO_4_ for 1 h, washed, and incubated in 2% aqueous uranyl acetate at 4°C overnight. After washing, cells were finally incubated in lead aspartate solution at 60°C for 30 min. After that, cells were washed, dehydrated with a series of washes in acetone, infiltrated and embedded in Durcupan resin.

Following polymerization, coverslips were removed with liquid nitrogen and hot water. Individual quadrants that had been imaged previously by confocal microscopy were trimmed, and serial sections were collected on slotted grids covered with formvar film and imaged by transmission electron microscopy (TEM). The cytoplasmic haze in the GFP channel and DAPI staining of nuclei were used to overlay 2D TEM images with individual optical sections of the 3D stack gained by confocal microscopy.

### Quantification and statistical analysis

A minimum of at least three independent experiments were performed per experiment. Statistically significant estimates were obtained from *N* independent experiments as defined in the figure legends. Unless indicated otherwise, statistics were performed with a two-tailed unpaired Student's *t*-test, and the mean±standard error of the mean (s.e.m.) was plotted in each graph. The *t*-statistic (t) and degree of freedom (d.f.) were determined using GraphPad Prism software. When more than two experimental groups were compared to a control experiment, one-way analysis of variance was performed, followed by a Dunnett post-test using GraphPad Prism software. Differences in the mean values were considered to be significant at *P*<0.05 (*P<0.05, **P<0.01, ***P<0.001, ****P<0.0001).

## Supplementary Material

Supplementary information
