# Supplementary material for: A SEPT1-based scaffold is required for Golgi integrity and function
Source: J Cell Sci. 2019 Feb 1;132(3):jcs225557. doi: 10.1242/jcs.225557 (PMC6382012; doi:10.1242/jcs.225557)
Supplement: Supplementary information [file joces-132-225557-s1.pdf]

Table S1

Antibody	SOURCE	IDENTIFiER	IF	WB
SEPT1	Santa Cruz	SC-398586		1:200 / 1:500
SEPT1	This study	N/A	1:200	1:500
SEPT2	Sigma-Aldrich	HPA018481	1:200	1:5,000
SEPT6	This study	N/A	1:50	
SEPT6	Sigma Aldrich	HPA005665		1:1,000
SEPT7	This study	N/A	1:100	
SEPT7	Santa Cruz	SC-20620		1:500
SEPT9	(Diesenberg et al., 2015)	N/A	1:400	
SEPT9	abnova	H00010801		1:500
GM130	BD Bioscience	610822	1:200	1:100
GM130	Abcam	ab52649	1:200	
γ-tubulin	Abcam	ab11316		1:1,000
α-tubulin	Sigma-Aldrich	T5168	1:500	1:2,000
EEA1	BD Biosciences	610456	1:100	
Ergic-53	Santa Cruz	sc-398893	1:100	
COPI	(Faulstich et al., 1996)	N/A	1:50	
AP1	BD Bioscience	610386	1:200	
GGA1	Abnova	H00026088-B01	1:100	
Vps26	Abcam	23892	1:100	
CEP170	Invitrogen	41-3220	1:100	1:100
CEP170	Abcam	ab72505		1:200
LAMP-1	BD pharmingen	555798	1:200	
Golgin97	Invitrogen	A-21270	1:50	
TGN46	Abcam	ab50595	1:100	
GAPDH	Sigma-Aldrich	G8795		1:20,000
Golgin104	Sigma-Aldrich	HPA018019		1:400
Giantin	Biolegend	924302	1:400	1:500
Talin	Sigma-Aldrich	T3287		1:2,000
Myc	Santa Cruz	9E10	1:200	1:1,000
GFP	Clontech	632381		1:2,500

Journal of Cell Science • Supplementary information

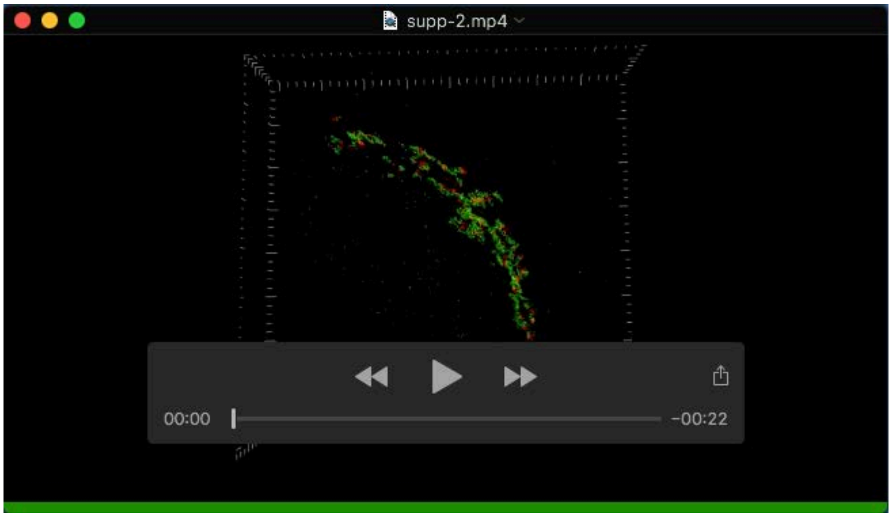

Movie 1. SIM control GM130green_ golgin97red

HeLa cells were treated with siCtrl, fixed, immunostained for GM130 (green) and golgin97 (red) and monitored by 3D-SIM microscopy.

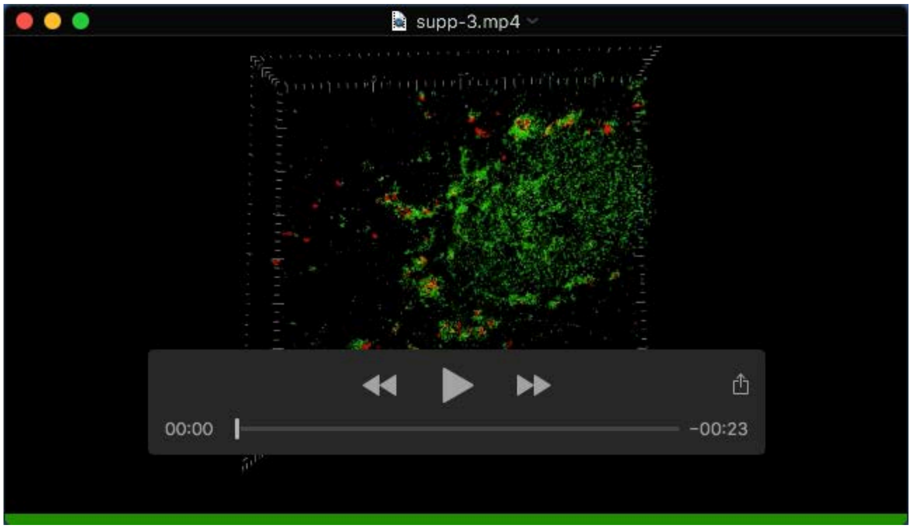

Movie 2. SIM siSEPT1 GM130green_ golgin97red

HeLa cells were treated with siSEPT1#1, fixed, immunostained for GM130 (green) and golgin97 (red) and monitored by 3D-SIM microscopy.

Figure S1

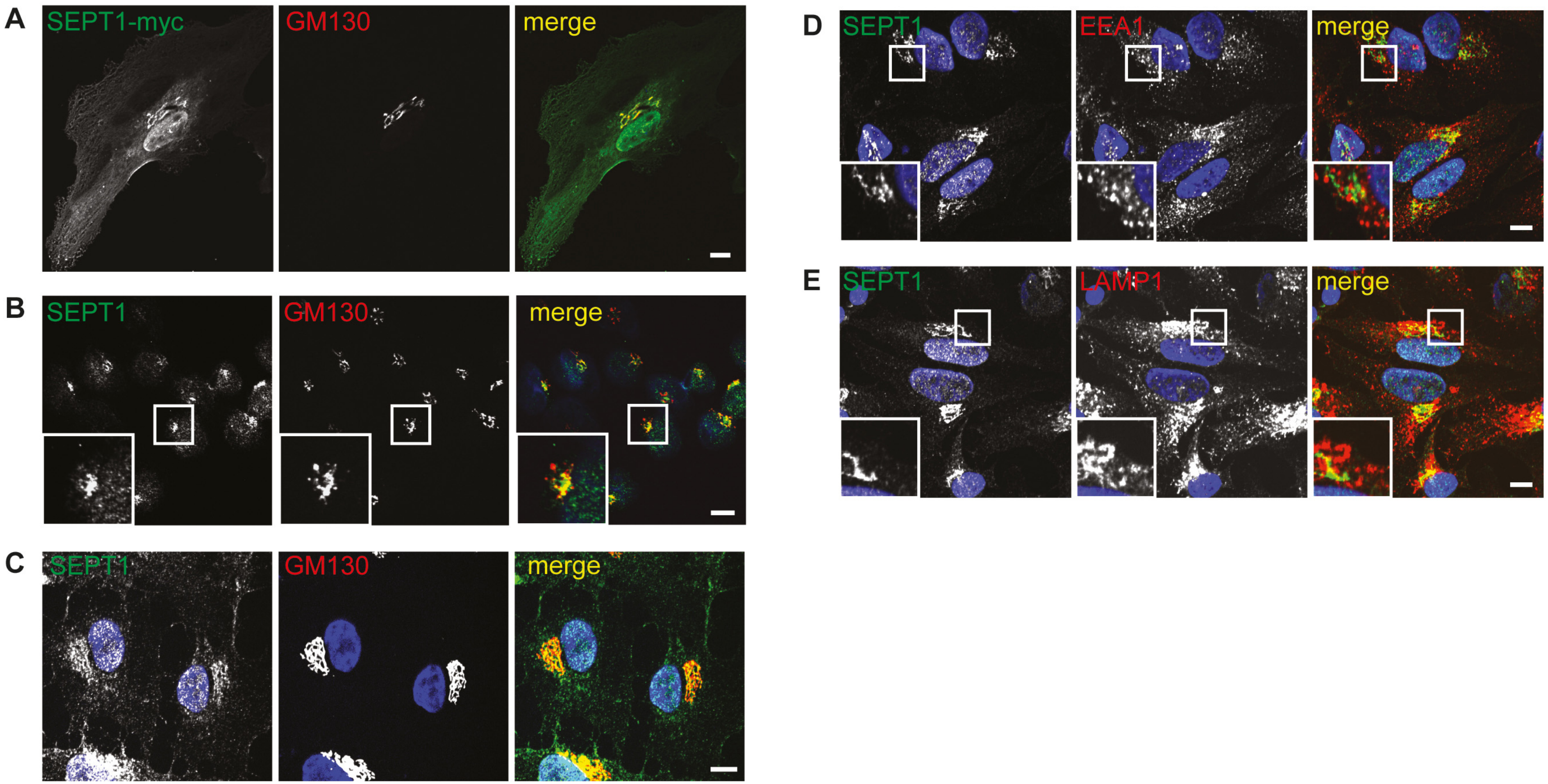

SEPT1 localizes predominantly to the cis-Golgi.
(A) Localization of myc-tagged SEPT1 upon overexpression in HeLa cells, and immunostaining for myc and GM130.
(B) Immunostaining of endogenous SEPT1 and GM130 in Jurkat cells.
(C) Immunostaining of endogenous SEPT1 and GM130 in RPE1 cells.
(D) Staining of SEPT1 and EEA1 (early endosome) in HeLa cells.
(E) Staining of SEPT1 and LAMP1 (lysosome) in HeLa cells. All scale bars, 10 µm.

Figure S2

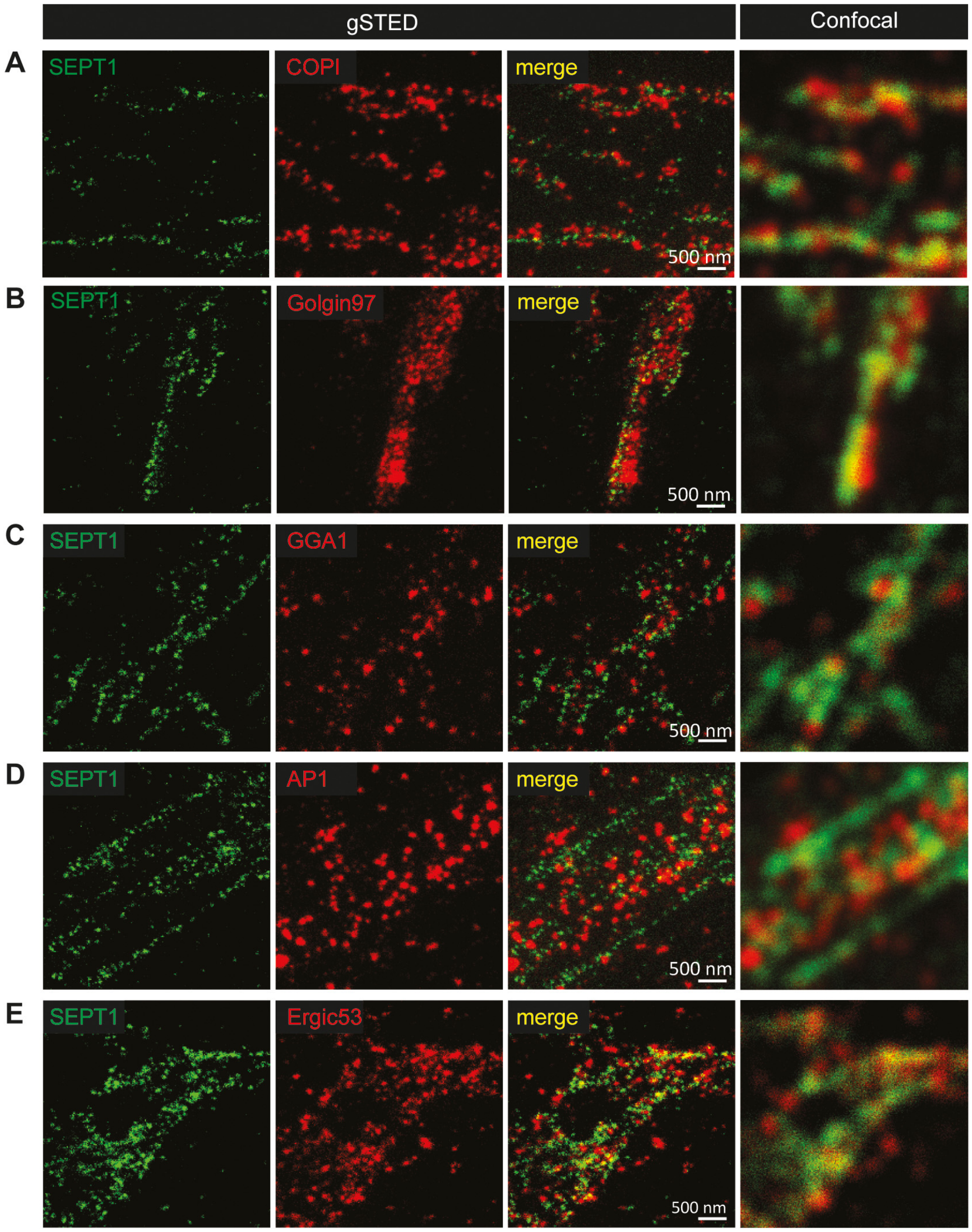

gSTED microscopy analysis of SEPT1 at Golgi membranes.
(A) Analysis of HeLa cells co-immunolabeled with antibodies recognizing SEPT1 and the trafficking adaptor COPI (recruited to the cis-Golgi and to ER-Golgi-Intermediate-Compartment, ERGIC),
(B) Golgin97 (trans-Golgi),
(C) GGA1 (trans-Golgi trafficking adaptor),
(D) AP1 (trans-Golgi adaptor), or
(E) Ergic53. All scale bars, 500 nm.

Figure S3

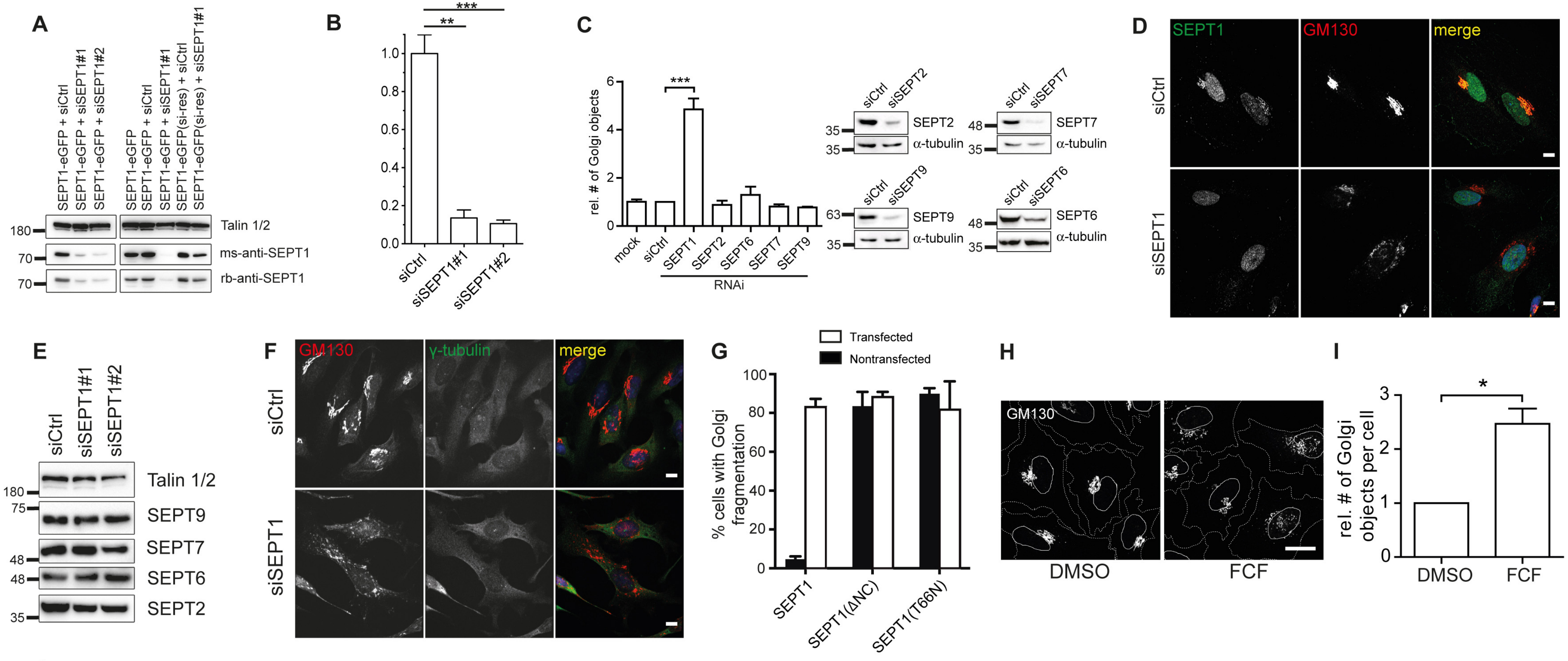

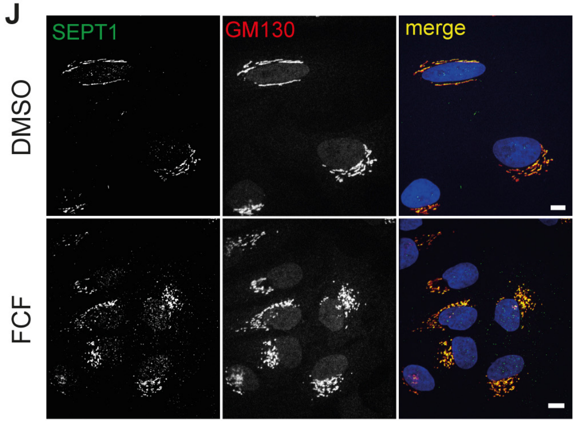

Depletion of SEPT1, but not of other septin family members perturbs Golgi integrity.
(A) Western blot analysis of lysates derived from HeLa cells upon co-transfection of SEPT1-eGFP and the indicated siRNAs.
(B) Quantification of SEPT1 mRNA levels determined by RT-PCR from HeLa cells, treated with the indicated siRNAs. Bars indicate mean (± s.e.m) SEPT1 levels upon normalization to actin B amounts. Unpaired two-tailed Student's t-test (SEPT1#1: N=3; mean=0.136; p-value=0.0013; t= 8.07; df=4; SEPT1#2: N=3; mean=0.106; p-value=0.0009; t=8.899; df=4).
(C) HeLa cells depleted of SEPT2, SEPT6, SEPT7 or SEPT9 were fixed and stained for GM130. Bars indicate the mean number of Golgi objects (mean ± s.e.m.). Unpaired two-tailed Student's t-test, ***P<0.001, SEPT1(N=6; t= 8.56; df=5), SEPT2 (N=3; p-value= 0.5581; t=0.6966; df=2), SEPT6 (N=3; p-value=0.4806; t=0.8596; df=2), SEPT7 (N=3; p-value=0.16; t=2.19; df=2), SEPT9 (N=2). Depletion of SEPT2, SEPT6, SEPT7 or SEPT9 was confirmed by Western blotting.
(D) Immunostaining for SEPT1 and GM130 in HeLa cells upon transfection of siCtrl or siSEPT1#1.
(E) Western blot analysis of lysates derived from HeLa cells upon transfection of the indicated siRNAs.
(F) Immunostaining for GM130 and □-tubulin in siCtrl- and siSEPT1#1-treated HeLa cells.
(G) Quantification of cells displaying a fragmented Golgi ribbon upon re-introduction of myc-tagged SEPT1 (FL), SEPT1 (□NC) or SEPT1(T66N) into siSEPT1#1-depleted cells.
(H) HeLa cells treated with DMSO or 150 µM FCF were immunolabeled for GM130. Scale bar, 15 µm.
(I) Quantification of Golgi fragments as depicted in (B). Data are represented as mean ± SEM, paired t-test, *P<0.05 (N=3; n=372-447; t=5.198; df=2).
(J) Immunostaining of SEPT1 and GM130 in DMSO- or FCF-treated HeLa cells. Scale bar, 10 µm.

Figure S4

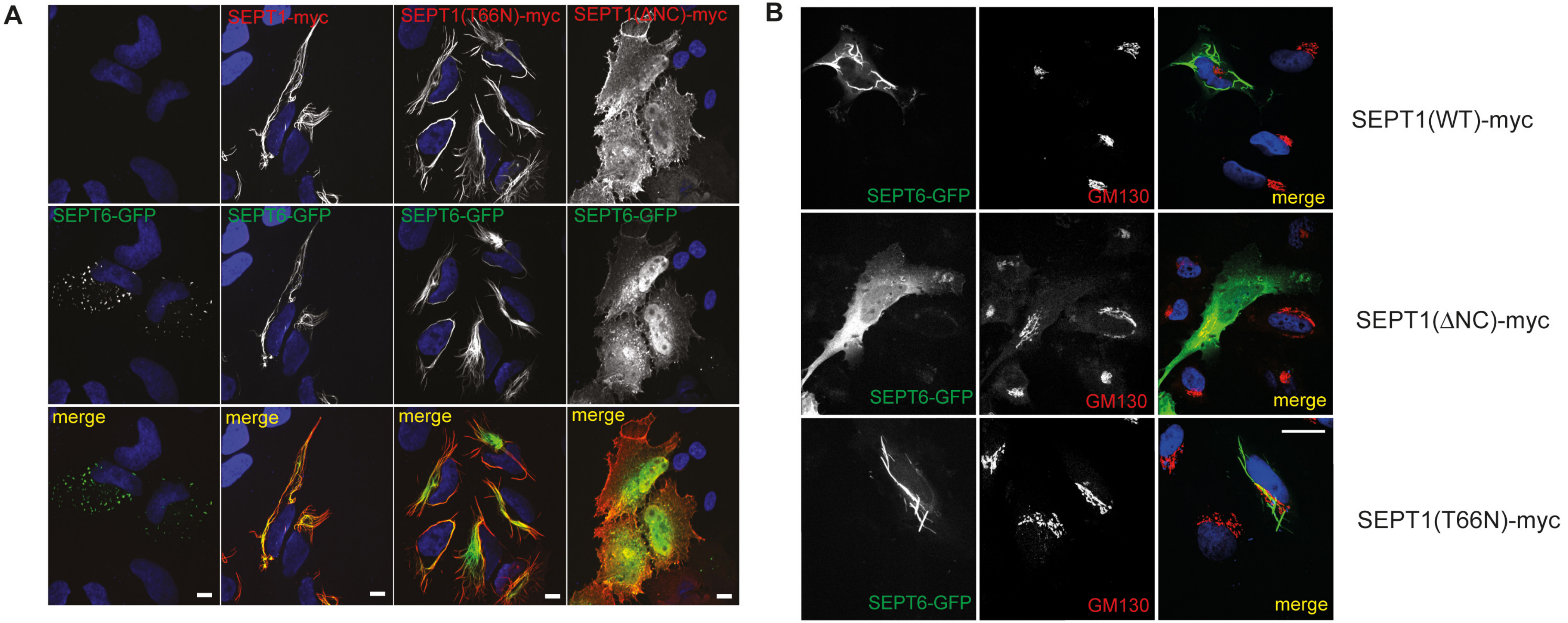

Analysis of SEPT1 variants used for rescue experiments.
(A) SEPT6-eGFP overexpressed alone, or co-expressed with SEPT1-myc (WT) or mutants. Co-expression with SEPT1(WT) or SEPT1(T66N) triggers formation of artificial septin fibers. SEPT1(ΔNC) cannot form filaments with SEPT6. Scale bar, 10 µm.
(B) Immunostaining of GM130 upon co-transfection of HeLa cells with SEPT1-myc (WT, ΔNC or T66N) and SEPT6-eGFP.

Journal of Cell Science • Supplementary information

Figure S5

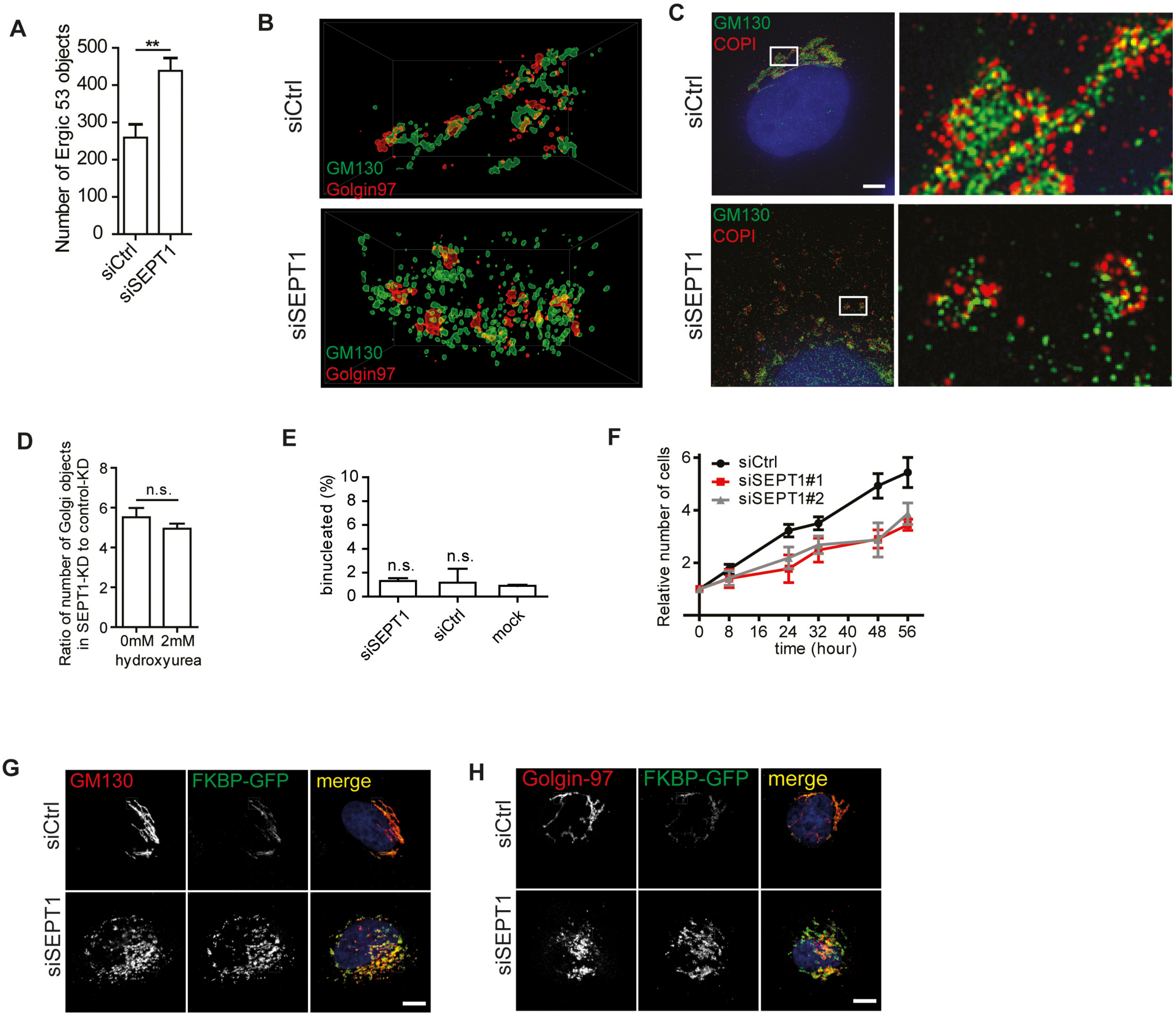

Analysis of Golgi defects in SEPT1-depleted cells by 3D-SIM, and of the impact of SEPT1 on mitosis, proliferation and secretion.
(A) Number of Ergic53-positive objects in control or SEPT1-depleted cells, as determined from 3D-SIM images. Data are represented as mean ± SEM, unpaired two-tailed Student's t-test, **P<0.01(n=9; t=3.623; df=16).
(B) Surface representation of cis- (GM130) and trans- (Golgin97) Golgi objects derived from 3D-SIM images.
(C) 3D-SIM image of GM130 and COPI labeling in HeLa cells after control or SEPT1-depletion.
(D) Relative increase in the number of Golgi objects observed in cells treated with 0 mM or 2 mM hydroxyurea (mean ± SEM). SEPT1 depletion increases the number of Golgi objects, and this defect is not cured by administration of hydroxyurea. Unpaired two-tailed Student's t-test, *P<0.05 (N=3; n=224-355; p-value=0.344; t=1.072; df=4).
(E) Percentage of binucleated cells in control and SEPT1-depleted cells (mean ± SEM), unpaired two-tailed Student's t-test, ns = not significant.
(F) Proliferation of HeLa cells transfected with control, siSEPT1#1 or siSEPT1#2 siRNAs and cells. Data indicate relative numbers of cells at different time points (mean ± s.e.m; N=3).
(G) Secretion of FKBP(F36M)-GFP reporter was induced for 30 min in control and SEPT1-depleted cells, and fixed cells were then immunostained for GM130 (cis-Golgi). Scale bar, 5 µm.
(H) Cells as treated in (G) were immunostained with Golgin97 (trans-Golgi). Scale bar, 5 µm.

Journal of Cell Science • Supplementary information

Figure S6

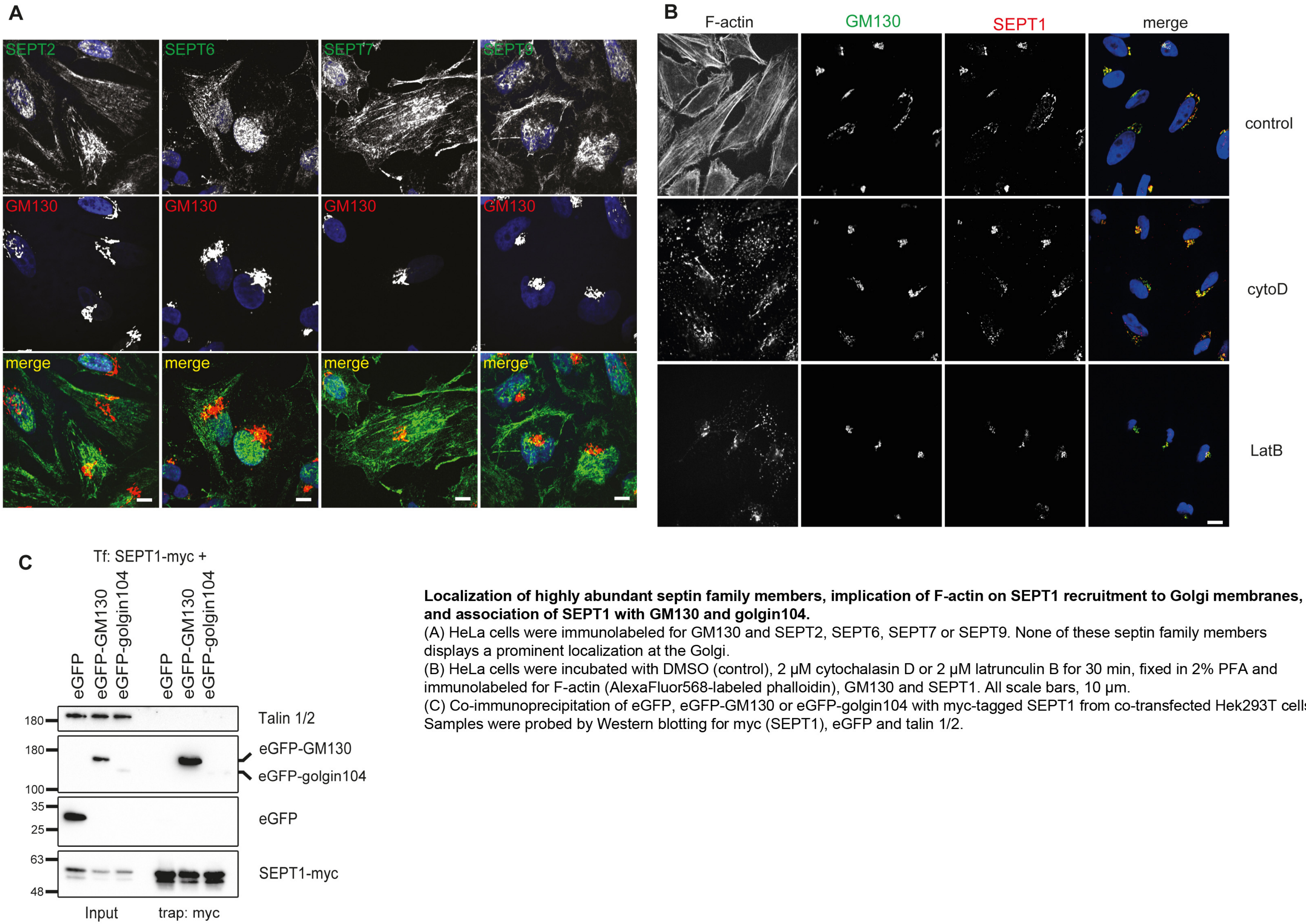

Localization of highly abundant septin family members, implication of F-actin on SEPT1 recruitment to Golgi membranes, and association of SEPT1 with GM130 and golgin104.
(A) HeLa cells were immunolabeled for GM130 and SEPT2, SEPT6, SEPT7 or SEPT9. None of these septin family members displays a prominent localization at the Golgi.
(B) HeLa cells were incubated with DMSO (control), 2 µM cytochalasin D or 2 µM latrunculin B for 30 min, fixed in 2% PFA and immunolabeled for F-actin (AlexaFluor568-labeled phalloidin), GM130 and SEPT1. All scale bars, 10 µm.
(C) Co-immunoprecipitation of eGFP, eGFP-GM130 or eGFP-golgin104 with myc-tagged SEPT1 from co-transfected Hek293T cells. Samples were probed by Western blotting for myc (SEPT1), eGFP and talin 1/2.

Journal of Cell Science • Supplementary information

Figure S7

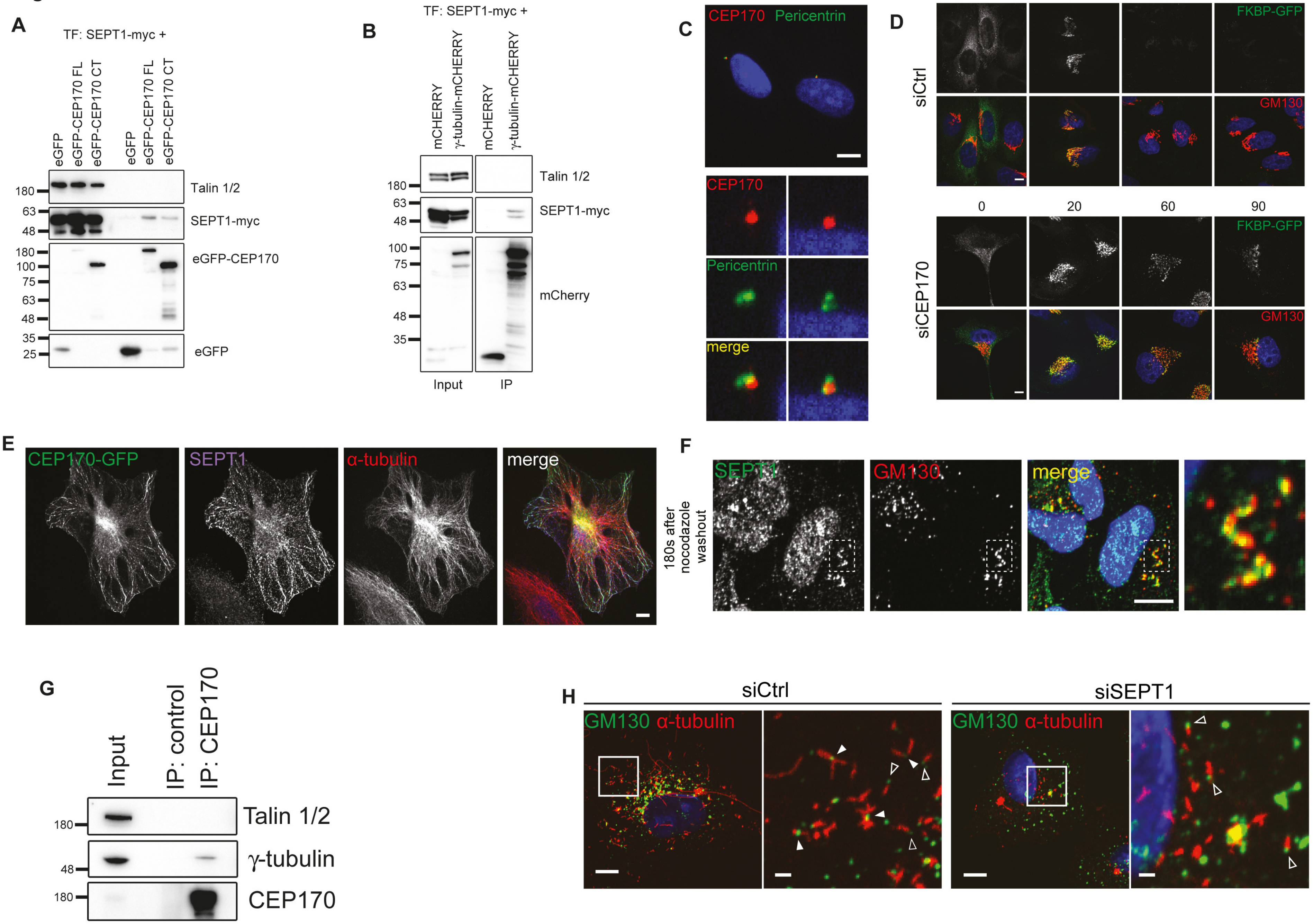

SEPT1 associates with CEP170 and γ-tubulin to connect cis-Golgi membranes to the microtubule nucleating machinery.
(A) SEPT1-myc co-purifies with eGFP-CEP170 full length, but not eGFP-CEP170 CT from co-transfected Hek293T cells. Samples were probed by Western blotting for myc (SEPT1), eGFP (eGFP), CEP170 (eGFP-CEP170 FL and CT) and talin 1/2.
(B) γ-tubulin-mCherry co-immunoprecipitates SEPT1-myc from transfected Hek293T cells.
(C) CEP170 localizes to the centrosome. Endogenous CEP170 and pericentrin were immunolabeled in methanol-fixed HeLa cells. Note that cold methanol disrupts septin filament integrity (not shown).
(D) Secretion of FKBP(F36M)-GFP reporter was induced for 30 min in control and CEP170-depleted HeLaM C1 cells, and fixed cells were then immunostained for GM130 (cis-Golgi) and monitored by confocal fluorescence microscopy.
(E) Upon overexpression in HeLa cells eGFP-CEP170 co-localizes with SEPT1 on microtubules.
(F) RPE1 cells were treated with nocodazole for 2 h, fixed in 2% PFA 3 min after wash-out, and immunolabeled for GM130 and SEPT1. Enlarged figure inset (right panel) depicts SEPT1 connecting neighboring GM130-positive Golgi objects.
(G) Endogenous γ-tubulin affinity-purifies together with CEP170 from Jurkat cell lysates. Endogenous CEP170 was immunoprecipitated, and the affinity-purified material was analyzed by Western blotting.
(H) Control and SEPT1-depleted RPE1 cells were fixed 90 s after nocodazole washout and stained for α-tubulin and GM130. Open arrows: single microtubule growing from an individual Golgi object. Closed arrows: grouped microtubules growing from an individual Golgi object. All scale bars, 10 µm.
